# Information-theoretic models of deception: Modelling cooperation and diffusion in populations exposed to "fake news"

**DOI:** 10.1371/journal.pone.0207383

**Published:** 2018-11-28

**Authors:** Carlo Kopp, Kevin B. Korb, Bruce I. Mills

**Affiliations:** 1 Data Science Research Group, Faculty of Information Technology, Monash University, Clayton, Victoria, Australia; 2 School of Mathematics and Statistics, University of Western Australia, Western Australia, Australia; Southwest University, CHINA

## Abstract

The modelling of deceptions in game theory and decision theory has not been well studied, despite the increasing importance of this problem in social media, public discourse, and organisational management. This paper presents an improved formulation of the extant information-theoretic models of deceptions, a framework for incorporating these models of deception into game and decision theoretic models of deception, and applies these models and this framework in an agent based evolutionary simulation that models two very common deception types employed in “fake news” attacks. The simulation results for both deception types modelled show, as observed empirically in many social systems subjected to “fake news” attacks, that even a very small population of deceivers that transiently invades a much larger population of non-deceiving agents can strongly alter the equilibrium behaviour of the population in favour of agents playing an always defect strategy. The results also show that the ability of a population of deceivers to establish itself or remain present in a population is highly sensitive to the cost of the deception, as this cost reduces the fitness of deceiving agents when competing against non-deceiving agents. Diffusion behaviours observed for agents exploiting the deception producing false beliefs are very close to empirically observed behaviours in social media, when fitted to epidemiological models. We thus demonstrate, using the improved formulation of the information-theoretic models of deception, that agent based evolutionary simulations employing the Iterated Prisoner’s Dilemma can accurately capture the behaviours of a population subject to deception attacks introducing uncertainty and false perceptions, and show that information-theoretic models of deception have practical applications beyond trivial taxonomical analysis.

## Introduction

The importance of models that accurately represent deception cannot be overstated. The pervasive use of digital communications, information storage and processing has led to a transformational paradigm shift much like that observed with the introduction of Gutenberg’s press in the fifteenth century [1, 2]. An unintended byproduct of this shift is the low cost incurred in exploiting data and information for deceptive purposes, resulting in a pandemic of deceptive behaviours, most recently, in social media. The problem is so pervasive, that a representative survey would be a major study in its own right, given several past studies each of much narrower scope [3–12].

The absence of a coherent and complete approach in how to best model deceptions has persistently impaired research that explores problems arising from the digital mass distribution of deceptive content, whether in social or mass media.

Many examples exist in which the absence of robust modelling methods has impaired understanding of empirically observed effects. An interesting recent instance was the widely reported case of the “Macedonian Fake-News Complex”, a *de facto* minor local industry formed by teenagers who were earning quite significant website advertising revenue by producing “Fake News”, and distributing it through social media during the United States presidential election of 2016 [13].

We define a deception as an action, or an intentional inaction, that aims to bring the second party to a false belief state, or to maintain a false belief state. The intent of a party producing a deception may or may not be to disadvantage the deceived party.

Framing the deception problem has presented persistent challenges in decision theory, and in game theory, in part due to the immense diversity and complexity observed in deceptive behaviours, and in part because deception is fundamentally an information-theoretic phenomenon, which impacts through its effects many problems in game theory and decision theory.

Li and Cruz aptly observed that “it is still difficult to directly formulate deception as an additional control input of a decision-maker in a real-world conflict situation. Questions of when and how to formulate deception practically remain illusive” [14]. This reflects the observation of Vane et al that decision theory and game theory share a focus on utility, but diverge in the area of probabilities and information, insofar as decision theory favours the use of knowledge about an opponent and explicit probabilities, to maximise utilities, while game theory favours perfect information and minimising vulnerabilities [15].

Deception impacts subjective probabilities of players, subjective utilities of players, or decision mechanisms, by means of hiding information, introducing uncertainty, introducing false beliefs, or changing how a player might interpret a situation. In many ways the problem of deception challenges assumptions commonly used in modelling problems using purely game theoretic or decision theoretic methods.

Deception aims to produce suboptimal strategies, utilities or choices in the cognitive system of the victim.

There is no shortage of literature, especially in the humanities, which empirically documents, analyses or taxonomically categorises human deceptions in social systems. The result of this is that most if not all deceptive games played in social systems are well known and understood. Notable studies are the works of Haswell and Heuer, dealing with military and intelligence deceptions, the works of Bernays and Goebbels, dealing with propaganda, political and sales deceptions, Berne’s studies of psychological games, a much more recent survey by Fleming and Zyglidopoulos exploring deceptions inside organisations, and Pettit’s study of the history of deception in commerce [7, 16–19].

Robust work dealing with the empirical study of deceptions can also be found in the behavioural and social sciences, psychology, and in areas such as computational linguistics, but extant research on deception in the decision theory and game theory communities remains sparse, reflecting the absence of widely accepted models for understanding deception [3, 5, 16, 20, 21].

Most often, the focus has been on how specific deceptions alter specific games and decision processes, rather than the manner in which the deceptions are produced. There is a recurring focus on the effects of deceptions, rather than the fundamental nature of the deceptions. Attempts to explain deceptions with wide generality have been few.

Greenberg studied deceptive game strategies from the perspective of decision theory and payoffs, explaining the motivation for deception [22, 23], while Li and Cruz explored the problem of conditions required for deception to produce effects in games [14].

Hypergames, as defined by Bennett, are another construct used in modelling deceptions. These are games of incomplete information, capturing a decision model, in which the players may not be fully aware of the nature of the game they are playing, or indeed that they are actually participating in a game. Bennett’s hypergame emerged following the initial work of Thompson and Spencer on games of deception [24], and exists in both ordinal and cardinal forms [25–30]. The ‘perfect information’ and ‘complete information’ assumptions do not hold for a hypergame. False beliefs, such as misperceptions, deceptions and surprise apply [30]. As with other extant game and decision theoretic constructs, the deception effects are integrated into the model, by altering player perceptions and outcome preferences in the hypergame.

Ettinger and Jehiel also focus on player beliefs, aiming for a general model of deception, and explain deception in games within the framework of social psychology [31, 32]. Guala’s philosophical criticism of game theory is that it suffers from “empirical anomalies”, which are argued to derive from players’ perceptions of games, choices in games, preferences and utilities in games [33]. The central argument underpinning “psychological game theory” is that player beliefs are central to human behaviours in games, and Geanakoplos et al argue that “…the traditional theory of games is not well suited to the analysis of such belief-dependent psychological considerations as surprise, confidence, gratitude, disappointment, embarrassment, and so on” [34].

The information-theoretic model of deception is centred in how false beliefs are produced. It was independently constructed in 1999 by Borden and by Kopp. Borden was initially solving problems in electronic warfare, while Kopp was attempting to explain common deceptions observed in social systems and the cyber domain. Both arrived at the same model, with some differences in nomenclature and scope [35, 36]. Later work by Kopp mapped a wide range of known deceptions in social systems into this model [37, 38], while Mills and Kopp mapped the model into known biological deceptions [39], and Brumley, Kopp and Korb studied the manner in which deceptions impact cognitive and decision cycles [40–42].

This model is now established in the information warfare community [43–45], as it provides a fundamental mathematical theory that can be easily mapped into well established models for electronic information transmission [46].

In this paper, we present a unified framework for modelling deception based on information-theoretic models, and apply this approach to demonstrate, in a simulation, two examples of how deceptive effects can disrupt social systems.

The main contributions of this paper are: 1) a survey and discussion of prior research in the area of information-theoretic modelling of deceptions, and in effects-based game and decision theoretic representations of deception; 2) the introduction of a more exact formulation of the information-theoretic *Corruption* model based on information-theoretic similarity; 3) mapping the information-theoretic models of deception into the decision theoretic model of Greenberg, and the derived game theoretic model of Li and Cruz; 4) introducing a new and general theoretical framework for modelling deception, combining information, game and decision-theoretic models; 5) demonstrating the use of the general framework by simulating two aspects of the “Fake News” problem using the Iterated Prisoner’s Dilemma game; 6) by analysis of simulation results, showing the high sensitivity of deceptions to the cost incurred by deceivers, and how even a very small number of deceiving agents can produce a large effect in a population.

The importance of the first four contributions is that they provide a systematic and coherent method for representing and modelling deceptions, which can be employed in simulations of social and other systems subjected to deception attacks. The importance of the fifth contribution is a model of how deceptions work in social media, validating the qualitative observation of the importance of costs against payoffs in deceptions, both problems that to date have been poorly understood [47].

## Methods

### The four information theoretic deception models

The information-theoretic models of deception are derived from two important ideas in information theory, specifically Shannon’s idea of channel capacity and the notion of information-theoretic similarity between two messages. A brief outline of these two concepts is included in Appendix 1.

In the Borden-Kopp model of deception [48], four information-theoretic models are defined, *Degradation*, *Corruption*, *Denial* and *Subversion*, each of which is a specific form of altering the victim’s perception.

Two of these models involve manipulation of terms in Shannon’s channel capacity equation, one model involves manipulation of similarity, and one model involves the manipulation of internal information processing methods, effectively by altering some internal algorithm or process in the victim system.

The different labels employed for the models in early publications reflect the different paths Borden and Kopp followed in identifying the model initially, and should be properly considered as descriptive mnemonics for identifying the respective models. We do not use the labels employed by Bell and Whaley [3, 5]. Abbreviated labels based on Borden’s nomenclature for the first three models are employed in this paper, as Borden’s model conflates the *Denial* and *Subversion* models under *Denial* [42].

A player can apply any number of the four models, concurrently, or separately, to change the opponent’s perceptions to gain an advantage [49].

The *Degradation* deception model conceals or hides information in noise, or other background messages, to introduce uncertainty or a false perception in a competing player’s belief. This model exists in overt (active) and covert (passive) forms. In the overt form, the deceiver produces the noise signal with sufficient magnitude that it prevents the victim from reliably recognising arriving information, but alerting the victim to the fact that it is being attacked [35, 39].

In the covert form, the deceiver aims to make the message indistinguishable from the background noise of the environment.

An overt *Degradation* deception amounts to manipulating the noise term in Shannon’s capacity equation, such that *N* ≫ *S* and in turn *C* → 0, while a covert *Degradation* deception amounts to manipulating the signal term in Shannon’s capacity equation, such that *S* ≪ *N* and in turn *C* → 0.

Camouflage, concealment and hiding are covert forms of this model. Flooding a victim with non-sensical, redundant or irrelevant data to hide actual facts are overt forms of this model.

The *Corruption* deception model produces a false belief by replacing a real message with a similar, but false message, contrived to be very difficult to distinguish from a real message. The false message thus mimics a real message. Successful corruption is inherently covert, as the victim remains unaware that the information is misleading [35, 39].

A *Corruption* deception amounts to fabricating a deceptive message sufficiently similar to a real message, that the victim cannot recognise the difference, so *S* → 1 inside the victim’s cognitive system, where *S* is information-theoretic similarity. Any deception in which a falsehood is contrived to mimic a truth is represented by this model. An improved formulation based on information-theoretic similarity is described in Appendix 1.

The *Denial* deception model increases uncertainty by preventing the victim from collecting information by disrupting or damaging the means employed to collect information. This model is always overt, as the victim is aware that the means has been denied, either in a temporary or persistent manner [35, 39]. A *Denial* deception amounts to manipulating the bandwidth term in Shannon’s capacity equation, such that *W* → 0, yielding in turn *C* → 0.

A card player seating himself in front of a brightly lit window, so that his opponent cannot easily read any tells, would be an instance of this model. Denial of service attacks in the cyber domain are another instance of this model.

The *Subversion* model involves actions where the victim’s information processing method or algorithm is altered to the advantage of the deceiver. This model is commonly employed for deceptions, but also is employed by parasites to compromise the basic objectives pursued by the victim. Most known instances of *Subversion* are combined with an initial *Corruption* attack to first insert the self-destructive message into the victim’s cognitive or decision cycle [39].

Some of the best illustrations of *Subversion* are political or commercial deceptions using “spin”, where the victim is encouraged to change the manner in which they interpret a message, to the advantage of the deceiver. It is important to note that *Denial* via *Subversion* can be employed for purposes outside the scope of altering perceptions.

**Fig 1:**
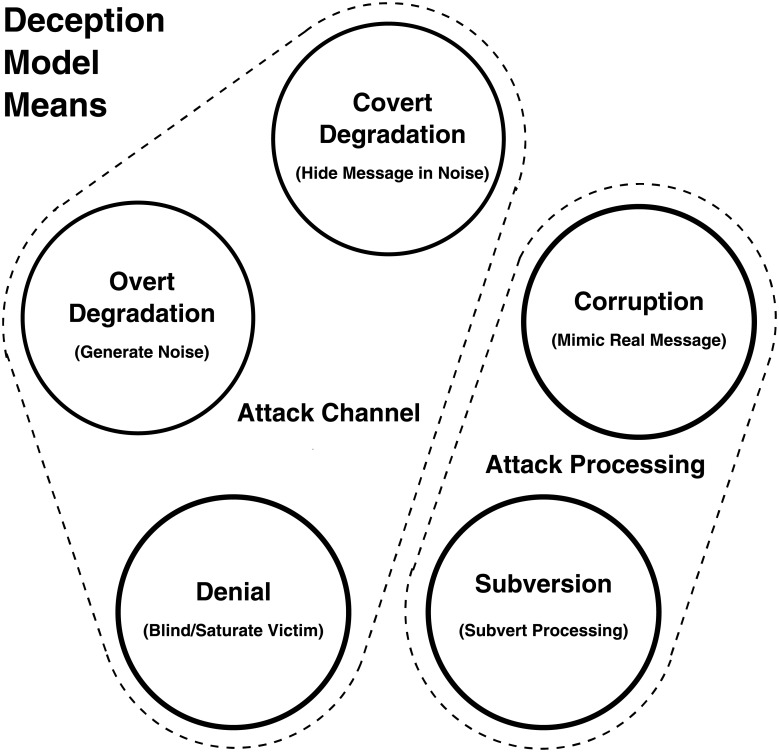
The means of executing the four information-theoretic deception models. The *Degradation*, and *Denial* deceptions attack the means of collecting information, which are the communication or perceptual channels. *Corruption* and *Subversion* attack the means of perception or processing, respectively.

Fig 1 depicts the relationships between the deception models and the components of system they are employed to compromise.

**Fig 2:**
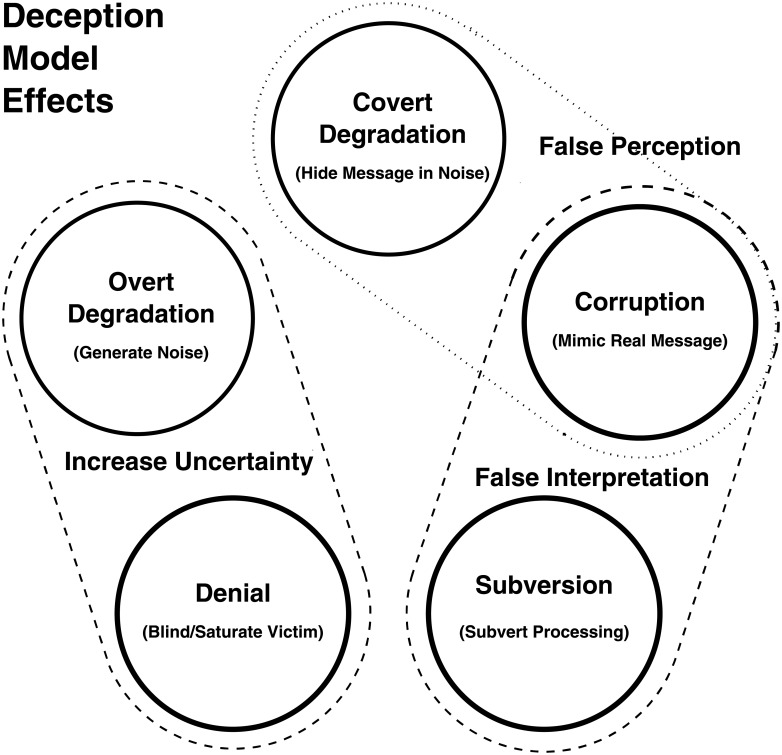
The effects of the four information-theoretic deception models. The deception effect of increased uncertainty is produced by the overt form of *Degradation*, and by *Denial*. False beliefs resulting from false interpretation or false perception are produced by the covert form of *Degradation*, *Corruption* and *Subversion*.

Fig 2 depicts the respective relationships between the deception models, when employed to produce deception effects.

Earlier studies have shown the ubiquity of these models in all domains where information is employed to gain a competitive advantage. A large number of empirical instances of known deceptions across the domains of social systems, cyber and biology were tested against this model, and invariably found to map into one of the four models, or some combination of these [37, 38, 40, 42, 46, 50].

The four canonical models have a number of interesting properties. The first of these is analogous to atomicity, in the sense that since each involves manipulation of different parameters, there can be no simpler models for a player to employ [49].

The second interesting property is analogous to orthogonality, and arises because these models can be applied separately, or in arbitary combinations, by an attacker. The latter leads inevitably to compound deception models, where the victim might be subjected to multiple serial and parallel deception attacks, the intent of which is to drive the victim into a pre-determined internal state desired by the attacker. A compound model can then be modelled as a directed graph, in which states of belief in the victim are represented as vertices, and deceptions which change the state of belief as arcs in the graph [37].

### An integrated framework for modelling deception

The central problems in games with deceptions arise from how the deception alters the victim’s beliefs, and how this in turn alters the victim’s decisions. The alteration of a belief is the effect produced by a deception. The information-theoretic models of deception map the deception into an effect.

Greenberg studied the problem of how deceptions impact players’ decisions in games. In Greenberg’s model a rational player will make the choice that maximises payoff, according to the subjective probabilities of payoffs for specific actions. These probabilities are derived from observations and prior beliefs, either or both of which may have been altered by a deception [23].

What the information-theoretic model of deception shows is that a player must make decisions when interpreting perceived inputs, before these inputs can be incorporated into the subjective model of the game, to estimate payoffs and risks, and to make decisions in the game. Decisions about perceived inputs are typically embedded in the perceptual and information processing mechanisms of the player, the behaviours of which are non-ideal and may or may not be readily altered by the player.

All four information-theoretic deception models are designed to defeat the mechanisms used by a player to develop correct or reasonable beliefs, which are subsequently employed to construct a payoff matrix for a decision, with the caveat that a *Subversion* deception may also alter the manner in which a player makes a decision, or acts upon the decision, as it may also alter utilities or decision algorithms.

The covert *Degradation* model is intended to transform a game of complete information into a game of incomplete information, by hiding facts, options or possible strategies from the victim.

The overt *Degradation* and *Denial* models are intended to introduce uncertainty into the victim’s decision process, to reduce the quality of the victim’s subjective probability estimates of payoffs or risks for specific actions. The victim knows that a deception is under way, but degraded or denied information produces uncertainty.

The *Corruption* model, and many instances of the *Subversion* model, are intended to introduce false beliefs by replacing facts, options or possible strategies with contrived alternatives, to the advantage of the attacker.

Seamless integration of the four information-theoretic deception models into the established game theoretic and decision theoretic constructs employed to model incomplete information, uncertainty, and false information, does present some practical challenges, mostly due to the immense diversity empirically observed in complex compound deceptions, the challenges of mapping perceptual models into subjective probabilities, but also due to the diverse foci in game and decision theoretic models, which may be oriented to understanding the strategies available, the specific decision, utility, payoff and risk models, or the possible equilibrium states, or lack thereof.

**Fig 3:**
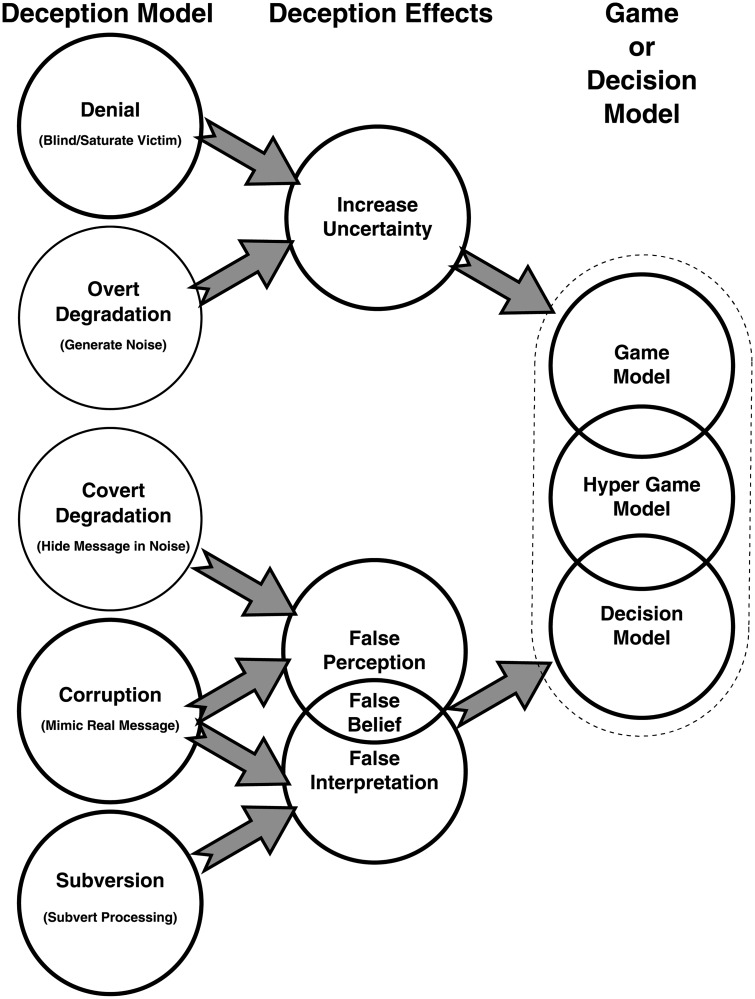
Deception framework. The mapping of deception models into deception effects for use in game and decision models. This mapping provides an integrated framework for modelling deceptions.

Fig 3 depicts the integrated framework for modelling deceptions. The deceptions produce effects, and these effects are employed as inputs to game or decision models.

#### Mapping the greenberg model

The study of deception in decision theory by Greenberg focusses on the effect of deception on players’ subjective probabilities in a decision matrix, and how this alters respective expected utilities or payoffs for specific alternatives or outcomes, but constrains the discussion of specific deceptions to a short qualitative survey, reflecting the foci of the study, and identifying the distinction between “false signals” and “noise”, which are effects that can be mapped directly into the four information-theoretic models [22].

The information-theoretic covert *Degradation*, *Corruption* and *Subversion* models map directly into Greenberg’s *False Signal* model, which captures the perceptual effects of these deception models, although conflating false perceptions and false interpretations into “false messages”. The overt *Degradation* and *Denial* models map directly into Greenberg’s *Noise* model, introducing the perceptual effects of uncertainty.

The information-theoretic models and Greenberg’s decision theoretic model are wholly coherent, through common albeit conflated deception effects in the Greenberg model. The principal challenge, identified by Greenberg, is in determining or estimating the changes to victim’s subjective probabilities resulting from the effects of the deception. That is inevitable, as such determinations or estimations reflect the specific manner in which a victim perceives and interprets the environment. In other words, how different kinds of deception affect different kinds of victim is an empirical problem.

#### Mapping the Li and Cruz model

The more recent study of deception by Li and Cruz defines two forms of deception, labelled “passive deception”, in which uncertainty is introduced by noise or randomisation, and “active deception”, in which a deceiver will “generate deceptive signals of high fidelity containing biased information to mislead its opponent” [14].

This work is a game theoretic remapping of Greenberg’s decision theoretic model, rigorously identifying constraints and conditions, and impacts on game strategies. The “passive” and “active” deception classifications directly map into the effects-based classifications used by Greenberg, and are thus equally coherent with the information-theoretic model, but also conflate false perceptions and false interpretations into false messages, labelled as “active deception”.

#### Mapping Bennett’s hypergame

Previous work studied how the four information-theoretic deception models can be integrated into Bennett’s hypergame, as this model provides a mechanism to capture the subjective perceptions and understanding of a complex game, as seen by the respective players [49]. We employed Bennett’s ordinal form, accepting that in some situations, a cardinal representation of preferences, favoured by Vane, may be more useful [15, 51, 52].

Applying the information-theoretic deception models to a second level Bennett hypergame yielded interesting results. The overt *Degradation* and *Denial* models introduced uncertainties into the perception of the opposing player’s subgame. The covert *Degradation* deception mapped into the strategic surprise variants of the hypergame, where opposing player strategies were hidden. False perceptions and interpretations induced by passive *Degradation*, *Corruption* and *Subversion* altered player’s understanding of the opponent’s subgame, and the opponent’s preferences [49].

We found that Bennett’s hypergame was the construct that provided the most flexibility in capturing the richness of the information-theoretic deception models. The multiple channel Normandy Invasion deception, commonly used as an example in hypergame studies, provides good illustrations of the information-theoretic deception models in multiple areas [53].

### Experimental modelling of the “fake news” problem

The term “fake news” is the most widely accepted label for the empirically observed problem of the mass distribution of deceptive content across mostly digital media. Wardle aptly describes this term as unhelpful, as its conflates misinformation and disinformation of various forms in various media. The “Misinformation Matrix” defined by Wardle respectively maps seven means and eight motives for the production and distribution of misinformation, based on empirical observation of social and mass media “fake news” [47].

Lazer et al defined “fake news” as “fabricated information that mimics news media content in form but not in organizational process or intent.” and observe that “Fake news overlaps with other information disorders, such as misinformation (false or misleading information) and disinformation (false information that is purposely spread to deceive people).” [54]

Campan et al defined “fake news” in a manner closer to Wardle, mapping it into categories of *clickbait*, *propaganda*, *commentary/opinion*, and *humour/satire*, dividing it further into *mis-information*, where the propagating party is unaware of the falsehood, and *disinformation*, where the falsehood is known to be false [55].

Notably, the motives for “fake news” production and distribution always involve some profit or gain by the players involved, be it monetary, political, ideological or psychological. Misinformation is mostly employed to produce specific effects in the victim audience.

Many instances fall under the common label of “clickbait”, the sole aim of which is generating monetary profit from web based advertising, regardless of other collateral damage effects inflicted on the hapless audience, such as producing a state of confusion. The deception is focussed on attracting attention and promoting or compelling further distribution of the misinformation, typically via social media.

Much more interesting are instances where the aim is political, ideological, or psychological, as the gain sought by an attacker is a change of perception or belief in the victim audience. Paul and Matthews explore this specific problem in a case study, and observe for that instance that producing confusion in the audience is now a common aim, counter to the past practice in political influence operations, “which traditionally emphasize the importance of truth, credibility, and the avoidance of contradiction”, the latter intended to introduce coherent beliefs in a victim audience aligned with the political or ideological agenda of the attacker [56]. The traditional practice is well studied, and has been previously mapped into the information-theoretic models [37, 38].

As the works of Wardle, Paul and Matthews, Lazer et al, and Campan et al show, the deceptions employed in “fake news” are predominantly of types that are represented by the *Degradation* and *Corruption* information theoretic models, with some of the more traditional propaganda constructs employing the *Subversion* information theoretic model. This is entirely consistent with earlier analysis by Kopp, studying empirical instances of political and commercial deceptions, where these three deception models were found to be most commonly used [37, 38].

Digital media with their inherent capability to amplify “fake news” traffic volumes add an additional dimension, as they facilitate “saturation” or “flooding” attacks, in which the victim is inundated with deceptive message traffic, possible via multiple channels. This type of deception attack was found to have multiple effects. Where the victim is unable to cope with the volume of messages, the effect becomes that of a *Denial* attack as the channel is effectively disabled; where the victim is able to cope with the volume of messages, the attack becomes an instance of overt *Degradation* where the deceptive messages are not coherent in content, and *Corruption* or *Subversion* where they are coherent in content [38].

Which of the four deceptions and respective effects are produced in the victim of a “saturation” or “flooding” attack depends on how the victim processes information. Does the victim ignore messages beyond some volume? Does the victim attempt to infer message veracity from the respective quantities of messages, employing the *argumentum ad populum* fallacy, and fall for a *Corruption* or *Subversion* attack? Does the victim simply become confused, suffering the effect of a *Degradation* attack?

To fully address the problem of “saturation” or “flooding” attacks in digital media requires a more complex model for a participant, in which different information processing schemas are employed to capture different types of “fake news” victim. However, each victim type will suffer effects defined by one or more of the four deception models, and therefore even a simpler study of the kind we conducted will provide some useful insights into the effects of such attacks.

Digital media are also characterised by message forwarding, such as “retweeting” or “sharing”, where victims of a deceptive message propagate the message to others, a model that fits the definition of a *Chained Compound Attack*, in which the party propagating the message becomes a proxy for the attacking party, knowingly or not [38].

To date most modelling effort dealing with messaging in social media has focussed on the diffusion of messages, mostly employing epidemiological models. Nekovee et al applied this approach to random and scale-free networks, relating propagation to network topologies [57]. A empirical study by Jin et al, using the SEIZ model developed by Bettencourt et al from the earlier SIR model, showed excellent agreement with *Twitter* social media traffic [58, 59]. Isea and Lonngren extended the SEIZ model to describe rumour propagation [60]. Zhao et al have studied other variations of the SIR model in rumour spreading [61–63]. More recently, Mussumeci and Coelhoa applied the SIR model to study the propagation of news [64].

Zubiaga et al studied empirically the behaviour patterns of social media users propagating rumours, showing that false rumours persisted longer due to the difficulty in debunking them, and showing that prevalent behaviour was to propagate rumours regardless of veracity [65].

The characteristic of the epidemiological models is that they capture diffusion behaviour, but are not intended to model the underlying mechanisms that determine the behaviour of the population, as an agent-based model is intended to do. For instance, in the SEIZ model, agents in a population can be *susceptible* or *infected*, and parameters in the model determine the rate at which the *infected* will infect the *susceptible* and the message diffuse through the agent population. In the SEIZ model, the population size remains unchanged, but the size of the *compartments* of the population comprising *susceptible* or *infected* agents change over time. The model cannot capture the internal causes for agents in the population to propagate a message as it was derived from epidemiology models intended to describe the dynamics of an infection, where infectious pathogens implicitly propagate themselves.

Recent studies by Petrov et al and Mikhailov et al used differential models to study the effects of propaganda on populations divided into groups choosing opposing viewpoints [66, 67], while Conover et al showed how intensive cooperation is central to the activity of online political communities [68].

Not well studied to date is the effect of “fake news” on cooperation in such communities. Political debate and voter choices often reflect consensus within a population on the suitability of competing political alternatives. Traditional political propaganda aims to alter beliefs, reinforcing consensus in supporters, and seducing the undecided and opposed, mostly employing *Corruption* and *Subversion* deceptions [37, 38]. Even less studied is the effect of political propaganda contrived to create confusion and thus uncertainty, using the *Degradation* deception. The effects of such confusion were recently explored by Flynn et al, who showed that misperceptions aligned with prior beliefs or agendas were more often accepted [10].

Comprehensive modelling of the whole gamut of effects empirically observed in “fake news”, especially in a contemporary digital environment, is a major challenge, due to the diversity of these effects, the presence of simple and compound deceptions, and the potential for different propagation topologies and social media participant behaviours.

Our experimental modelling explored the effects of the *Degradation* and *Corruption* deceptions on populations, emulating the two styles of political propaganda currently prevalent in social media. The experimental platform was an evolutionary Iterated Prisoner’s Dilemma (IPD) simulation, in which agents can evolve a range of well known IPD strategies, and constrained models of deception. The IPD was chosen specifically as it is widely understood, providing a good basis for comparisons and interpretation of results, and because it captures relative performance of cooperative and uncooperative strategies well [69, 70].

There are few studies that explore the effects of deceptions in populations using evolutionary simulations. Számadó et at in their study of the effects of deceptive messaging in an evolutionary simulation, employed a variant of Ohtsuki’s donor-recipient reciprocity model. This model is not an IPD, and randomly pairs donors and recipients, who can cooperate, defect or punish. Dishonest signalling was employed to manipulate victim perceptions of player reputations, in which good or bad reputations could be misrepresented as the opposite. In terms of the information theoretic models, this misrepresentation maps into the *Corruption* deception. Our experiment on the effects of *Corruption* differs in a number of respects, primarily in the use of a different game, and in the inability of players to identify other players by past reputations, as in our model players remember only their own experienced outcomes, and not the identity of the past opponents who produced them [71, 72].

A particular focus in our modelling was to assess the impact of the cost of deceptions on population behaviours, as this reflects a real world scenario, where the effectiveness of a deception may be improved by increased effort while incurring increased costs. We did not model variable deception effectiveness as a function of cost.

### Experimental design for agent based modelling of the “fake news” problem

The two series of experiments we conducted show the emulation of behaviours observed empirically in social media, by using an agent based IPD simulation with random pairing, but also show that information-theoretic models of deception can be employed in simulations to good effect.

The first experiment was designed to show that even a small fraction of agents in a population that conducts *Degradation* deceptions that introduce uncertainty can disrupt cooperation across a much larger population of agents, while also exploring the cost dependencies of these behaviours.

The second experiment was designed to show that in a population where agents conducting the *Corruption* deception are allowed to evolve and invade the population, this agent behaviour follows very similar diffusion behaviour within the population to that observed by Jin et al, Mussumeci et al and others in social media propagation of messages, while also exploring the cost dependencies of these behaviours.

Both experiments were thus designed to capture the propagation and amplification of deceptive messages, the effect of deceptive messages on an actively engaged population that is mutually interacting, and to explore sensitivity to the cost of deceptions.

The simulation was implemented using Netlogo *turtles* as agents [73]. We ran this simulation with a population of 50 agents, that is of the proper order of magnitude for Dunbar’s number in social media populations [74].

This is significant insofar as debates in social media typically involve smaller groups, with very much larger numbers of passive participants observing the debate. Campan et al studied “fake news” distribution mechanisms and identified a recurring practice pre-dating the digital social media, in which producers of fake news content target highly engaged and visible groups or individuals, who then become proxies that distribute the deceptive messages, thus implementing a *Chained Compound Attack* [55], [38].

Our initial assessment was that providing a population size of the order of Dunbar’s number should produce representative results for the social media context explored, as Dunbar’s number provides a reasonable bound on the size of a highly engaged and visible group in social media.

As the results of both experiments using this constrained population size demonstrated behaviours empirically observed in much larger real world environments, we considered that the additional effort in migrating the simulation to an environment compatible with much larger population sizes was not easily justified. It would be easily justified for more complex experiments capturing deceptive message propagation in complex and constrained network topologies. Given both the diversity and variability of social media and other environments subject to “fake news” attacks, the generality of the results will depend on how closely the environment resembles the types we compare our results against.

As the simulation is very computationally intensive, the population size of 50 required 14 days of computation time on the fastest four core processor available at that time, specifically an overclocked Core i7-6700K. Considerable effort was invested in profiling the performance of the simulation, which was constrained to four concurrent simulation runs. This effort showed that the Netlogo runtime environment Java Virtual Machine represented a serious performance bottleneck.

Because the computation time of the simulation scales with the square of the population size, simulating fully sized populations representative of real world social media environments in reasonable time was not feasible, without a different simulation environment that is suitable for a large parallel processing platform.

Agent reproduction in this simulation employs a two-point crossover at random locations, followed by a probabilistic mutation using an evolved mutation probability initially common to all agents.

In each generation, we remove the two agents with the lowest score from the population. We then select an agent probabilistically weighted on score and breed this agent with another randomly selected agent to produce two new offspring that replace the previously removed agents. This bounds the diffusion rate of invading strategies into a population.

The cumulative score determines agent fitness, and whether the agent will reproduce or die out. Agent fitness is a useful measure of effectiveness in modelling this problem area, as its effect can be mapped on to the popularity of “fake news” messages. If they are popular they propagate better thus increasing exposure of the victim audience to the deceptive message.

We employed the extant internal Netlogo *turtle* prisoner’s dilemma game that employs non-negative payoffs, which satisfy the prisoner’s dilemma condition of *T* > *R* > *P* > *S*, and can be remapped into the donation game parameters *b*, *b* − *c*:

- Temptation (*b*): *T* = 5
- Reward (*b* − *c*): *R* = 3
- Punishment: *P* = 1
- Sucker: *S* = 0

As the simulation employs a cumulative score as a measure of agent fitness, a form with non-negative payoffs is convenient as the fitness value is non-negative. We did not assess alternative values of Temptation and Reward in this simulation, as *N* such parameterizations would have increased required computational effort *N*-fold. Moreover, other parameterizations in the IPD would deny simple comparisons against earlier modelling using the default Netlogo IPD parameters.

The agent population was initialised with seven well known IPD strategies, specifically *TFT, TF2T, Pavlov, Always Cooperate, Always Defect, Random* and *Probabilistic*. The mix of strategies was intentional, to explore how the deceptions impacted susceptible strategies. In part this approach was also employed to provide control cases as some strategies, such as *Always Cooperate* and *Always Defect* will not be impacted by deceptions.

Each agent has memory to retain a history of three previous opponent moves, employed to determine its next move, given its evolved strategy.

The strategies differ in how they examine their history to select the next move. *Always Defect, Always Cooperate* and *Random* ignore the previous moves of the game. *Tit for Tat* considers only the opponent’s previous move. *Tit for Two Tats* considers the two previous observed moves. *Pavlov* considers the opponents’ previous move and the player’s previous move. *Probabilistic* considers all the previous moves of past opponents, choosing to defect or cooperate with equal probabilities to previously encountered opponents.

The cooperative strategies of most interest consider only the previous or two previous opponent moves, therefore additional depth in the history would not provide a benefit. This was confirmed by experimentation with early variants of the simulation that showed that increasing the depth of the memory to more moves, specifically four and five, did not appreciably alter results, but did increase simulation execution time appreciably, due to the high average frequency of operations involving alteration of agent memory. Therefore the final simulation was run with agents remembering only three previous moves, as this was assessed to provide sufficient sensitivity to deception effects in reasonable computation time.

For the experiments we report here, we ran the simulations in both sets of experiments for 5001 time steps. To provide control cases, some simulations were run in the first experiment with an initial population of deceiving agents, and some were allowed to evolve deceptions.

#### Simulation agents and globals

Each agent has defined characteristics:

- A mutation probability
- An inherited *S* strategy
- An inherited *D* deception method, or none, initially set by parameter
- A history showing the last three opponents’ moves
- A cumulative score

Additional parameters are buried in the code as global variables, since we had little need to adjust them once reasonable values were found. These include the cost of a deception that we parametrised, the Gaussian variance for mutation, and initial population size.

We detail simulation parametrisation and outputs in S1 Appendix.

#### Integration of the deception models

Of the four possible deception models, three are feasible in the IPD. The information theoretic *Denial* model is inherently incompatible with the IPD, as players employing information theoretic *Denial* signal their uncooperative intent to victims implicitly, rendering the method ineffective, as the victim will always know it should defect.

The *Subversion* deception results in the victim’s strategy being changed permanently to *Always Cooperate* and is not explored in this study, due to the additional complexity of designing a simulation that first primes a victim population by the use of *Corruption* to make it susceptible to a *Subversion* deception, which is the most common pattern observed empirically in social systems [37].

Deceptions involving large scale “flooding” or “saturation” attacks against a population are also not explored in this study. This was due to the inherent incompatibility of the information theoretic *Denial* model with the IPD game, and the previously discussed complexity of modelling the variability in effects upon a victim population, that may encompass *Denial*, *Degradation*, *Corruption* and *Subversion*. The experiments we did conduct do capture the effects of *Degradation* and *Corruption* deceptions that may arise in a “flooding” or “saturation” attack, but without a model that captures the statistically variable population fractions susceptible to the effects of the respective deception types.

As agents do not signal individual identities to other agents, an agent cannot associate a specific history of prior use of a deception method with another agent. A deception operates on a victim agent by altering the victim agent’s history or strategy before the IPD is played in a manner that captures the effect of that deception method, while the victim agent’s actual payoff reflects the actual strategy of the attacking agent. The attacking agent’s payoff is the game payoff less the *Cost* of the deception employed. Agents that deceive will perform the deception on every iteration of the game.

- *Degradation* replaces the three most recent moves in the victim agent’s observed history of its opponents with random moves
- *Corruption* overwrites *Defect* moves in the victim agent’s observed history of its opponents with *Cooperates*
- *Denial* prevents the victim agent from observing the attacker’s move for a single round, but was not employed in these simulations
- *Subversion* will set the victim agent’s strategy permanently to *Always Cooperate*

In practice a deception may or may not produce an effect, compared to a situation where neither player is deceiving. The deception is successful if the payoff is greater than the payoff without a deception, and unsuccessful otherwise. Whether a deception produces an effect or not, the deceiving player always incurs the cost of the deception, which is subtracted from the deceiving agent’s payoff. This reflects the reality that unsuccessful deceptions are inherently damaging to the deceiver, and deceptions with weak or no effect may also do more damage to a deceiver than a play without a deception.

We addressed this by the use of the following model:

- *No Deception*: Player A and Player B do not deceive, unaltered IPD strategy outcomes are employed
- *Deception by Player A*: Player A is a deceiver, Player B is a non-deceiver, player A deceives and plays its IPD strategy (we record success or failure of deception), while for player B, the IPD strategy outcome is determined by the effect of the deception on its IPD strategy
- *Deception by Player B*: Player B is a deceiver, Player A is a non-deceiver, player B deceives and plays its IPD strategy (we record success or failure of deception), while for player A, the IPD strategy outcome is determined by the effect of the deception on its IPD strategy
- *Mutual Deception*: Both players A and B are deceivers. We calculate the respective payoffs for both players without deceptions and save the payoffs in a temporary variable. We apply the respective deceptions to the memories of both players, and then calculate the respective payoffs for both players with deceptions. We use the saved payoff values in the temporary variables to calculate the success or failure of the respective deceptions.

Common empirically observed instances of mutual deceptions often show outcomes where neither player gained from the deception, as both suffered reduced payoffs resulting from opponents’ deceptions.

We provide a more detailed description of this model, and examples in S2 Appendix.

In all the experiments reported here, means are reported for sets of 30 runs with common simulation parameters, with variation due only to the seed used for the pseudo-random number generator. We employed the internal Netlogo new-seed function that produces a seed within a range of -2147483648 to 2147483647. In initial simulation testing, we verified simulation repeatability across multiple host platform types by fixing the seed value.

We used Netlogo’s *Behavior Space* facility to systematically vary the simulation parameters, such as cost of deceptions across sets of runs. Cost of deceptions is an important parameter, as deceptions that on average yield poor outcomes and high costs reduce the fitness of agents.

### Experiments on the effects of degradation

The purpose of this set of experiments was to establish whether a small population of players introducing uncertainty into the memories of a large population of non-deceiving players could significantly alter the frequency of cooperative behaviours in the non-deceiving population. This experiment was in effect intended to explore the impact of confusing “fake news” being injected into a community of voters, who are intending to vote a particular way, and thus cooperate in public discourse by agreeing with each other to reinforce their subjective certainty in a particular voting choice. The introduction of confusing “fake news” has been claimed to produce dischord and increase the degree of uncertainty in voters making up their minds [56].

The problem of noise disrupting the TFT strategy, first described by Molander, has been well studied, but has been previously framed as a result of memory errors or decision errors, which are not correlated with player intent [75–77].

Our hypothesis was that the use of the *Degradation* deception to increase the level of uncertainty in decisions across a population of players using cooperative strategies such as TFT and TF2T would produce identical effects to the well studied problem of noise being injected into the decisions of agents employing strategies such as TFT. The experiment was intended to not only demonstrate this behaviour, but also provide measures of sensitivity to deception cost, the impact of the size of the population that is employing the *Degradation* deception, and the effect on strategies other than TFT and TF2T.

In defining this experiment, we considered the mapping of strategies in the simulation to the respective roles deceivers and victims of deceptions play in social media interactions. The behaviours of highly “polarised” participants will map into the *Always Defect* strategy, while the behaviours of consensus seeking participants will map into strategies such as *Always Cooperate*, and variants of *Tit-For-Tat*.

We parametrised cost across a range of values (0.05, 0.1, 0.15, 0.2, 0.25, 0.3), as earlier calibration runs of the simulation indicated that *Degradation* costs in excess of 0.2 were not sustainable in this simulation.

The populations for all simulations in this experiment were initialised with cooperative strategies, i.e. *Tit for Tat, Tit for 2 Tats, Always Cooperate, Pavlov* or *Probabilistic*, so we could observe non-cooperative strategies, i.e. *Always Defect* and *Random*, evolving and invading the population if their fitness permits this to occur. An agent playing any strategy can employ deception, with the consequence that strategies for which concurrent deception on average reduces payoffs will be unable to become established in the population due to reduced fitness.

### Experiments on the effects of corruption

The purpose of this set of experiments was to establish the manner in which a population of agents performing *Corruption* deceptions, given some cost, would expand into a larger population of agents that are not deceiving. This experiment was in effect intended to emulate the diffusion of “fake news” in social media, for a population of agents that can derive a payoff from the deception.

The “fake news” problem is characterised by agents who propagate a message to gain an implicit reward, for instance by subjective gratification, or an explicit reward by advertising revenues. If the message is not “liked” or not propagated further by “retweeting”, the agent does not earn a reward [47].

This represents, as noted earlier, an instance of the *Chained Compound Attack* in which the entity propagating the confusing message becomes a proxy for the entity conducting the deception. A victim of a deception becomes a proxy of the deceiver the instant this victim propagates the deceptive message, and whether the deception is actually believed by the victim might not matter.

Our evolutionary simulation cannot propagate a pathogen or a belief, but agents can produce offspring that propagate characteristic properties, comprising a combination of a strategy and the use or otherwise of a deception. Notionally this is a remapping of the problem of a population shifting between states into a birth-death process representation, in which deaths and offspring are used to represent a state change as occurs in an epidemiological model such as SEIZ. This permits capture of the diffusion behaviour observed in epidemiological models.

Unlike the SIR and SEIZ epidemiological models, the IPD simulation provides control of a wider range of parameters, especially the payoffs and costs to players in the population.

This is important as in social systems, imperatives for propagating a message, false or true, derive from payoffs to the players. Fitness to produce offspring becomes a proxy variable to capture the transition from uninfected to infected states in an epidemiological model. We were especially interested in the sensitivity of this simulation to the cost of the deception, given its importance in social systems.

To test the simulation results for the characteristic diffusion behaviours previously observed in social media message propagation we devised an algorithm to fit a differential epidemiological model akin to SIR [59, 61–64].

This model describes the dynamics in terms of only the population fractions exhibiting specific behaviours *s*_*i*_ of the entire agent population, in the manner of compartments in epidemiological models. Each population is assumed large enough that *s*_*i*_ is continous.

For a locally conserved population of identifiable agents, the birth, death, immigration, and emigration rates are set to zero. And conversions form a point process with a constant rate. Let *β*_*jk*_ be the per capita rate of spontaneous conversion from trait *i* to trait *j* and *μ*_*ijk*_ the per capita per meeting rate from *j* to *k* induced by a meeting with *i*.

$${\dot{s}}_{k}=\sum_{j}\beta_{jk}s_{j}+\sum_{ij}\mu_{ijk}s_{i}s_{j}$$

Given time series data [*s*_*k*_]_*t*_ where *t* is a discrete time of a discrete simulation. There is also ${\left[{\dot{s}}_{k}\right]}_{t}$ and [*s*_*i*_ *s*_*j*_]_*t*_ for each *i*, and *j*. The problem of model fitting is to determine ${\left[{\dot{s}}_{k}\right]}_{t}$ as a linear combination of the [*s*_*k*_]_*t*_ and [*s*_*i*_ *s*_*j*_]_*t*_. Given *m* agent behaviours there are *m* distinct [*s*_*k*_] and *m*(*m*+ 1)/2 distinct [*s*_*i*_ *s*_*j*_]. Place these in a matrix *F*. Let *U* = *F*^*T*^ *F*, almost always non singular, and $V=F^{T}\left[\dot{s}\right]$, the optimal parameters are $A=U^{-1}V={\left(F^{T}F\right)}^{-1}F^{T}\left[\dot{s}\right]$, and the differential estimation is $E=F^{T}A=F^{T}{\left(F^{T}F\right)}^{-1}F^{T}\left[\dot{s}\right]$.

Population decays were also fitted, to compare as a control case against other work on decay transients in social media traffic [78].

We include a more detailed discussion of the fitting method in S4 Appendix.

## Results

### Simulation results for experiments on degradation

Figs A-N in S3 Appendix show the evolution over time of the average populations of agents playing IPD strategies, parametrised by cost.

In assessing the equilibrium behaviours observed in an evolving population with a mix of different strategies, we employ the dynamical outcome descriptions employed by Le and Boyd in their study of evolutionary dynamics in a continuous IPD, noting that their simulations employed a population of 8,000—10,000 agents for 1,000 to 3,000 generations, while we employed a population of 50 agents for 5000 generations [79]. Le and Boyd identify four dynamical outcomes, labeled as *stable cooperative equilibrium*, *cyclical polymorphism*, *stable polymorphism*, and *collapse to a non-cooperative equilibrium*.

Other than a set of early simulation test runs intended as a control, the simulations were all initialised with an equal mix of cooperative strategies, allowing the exploitative *All Defect* strategy to randomly evolve and invade the population. There were no significant differences between the simulations initialised with a population of deceiving agents, and simulations initialised without deceiving agents. S1 Appendix presents data for simulations initialised without deceiving agents.

**Fig 4:**
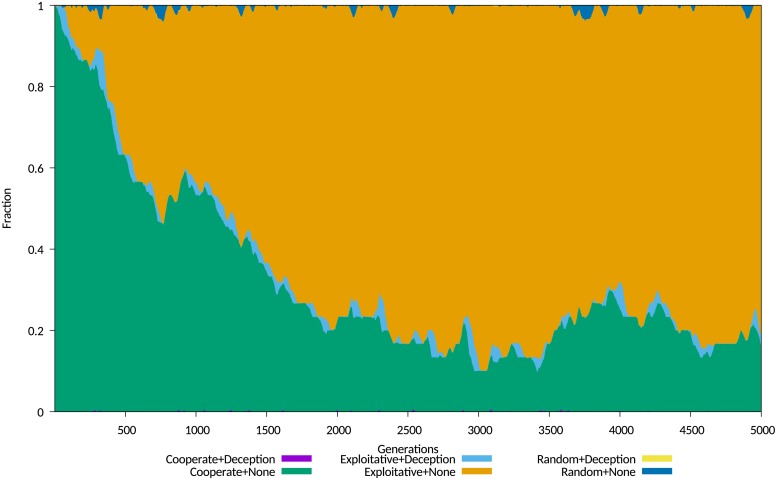
Population map for degradation experiments at low cost. Population map, with grouped cooperative and exploitative strategies, for Cost = 0.05.

**Fig 5:**
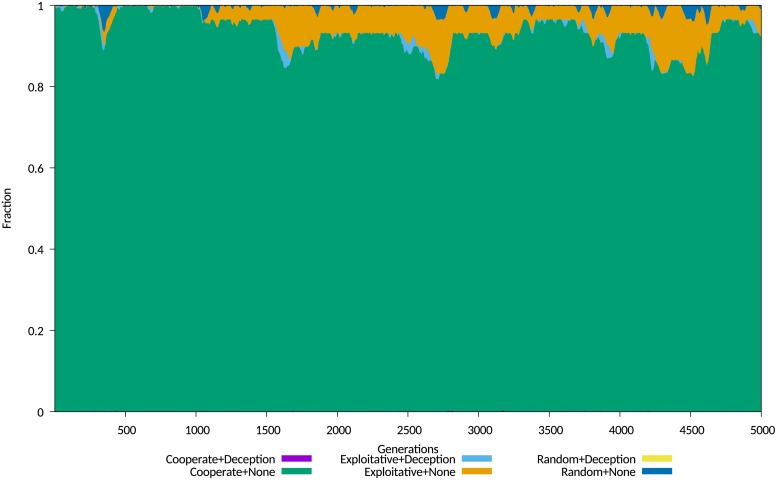
Population map for degradation experiments at high cost. Population map, with grouped cooperative and exploitative strategies, for Cost = 0.3.

The first set of plotted data show the average sub-populations of agents, grouping them into six sub-populations, determined by the use of IPD strategy and the use or non-use of the *Degradation* deception, with the *Random* strategy as a control. These sub-populations may be broadly described as either cooperative or exploitative, based upon their use or otherwise of a cooperative IPD strategy. Where a combination of a strategy and deception yields on average a low fitness, that combination extinguishes itself very quickly. Examples for a cost of 0.05 and 0.3 are shown in Figs 4 and 5 respectively.

The observed equilibrium behaviours fit the *stable polymorphism* identified by Le and Boyd, with an initial population of non-deceiving agents playing cooperative strategies invaded by a mix of deceiving and non-deceiving exploitative agents, with a stable but noisy equilibrium becoming established between 500 and 2,000 generations.

There is no evidence of periodicity in the equilibrium leading to the *cyclical polymorphism*, or a trend within the 5000 generation interval to a state of *collapse to a non-cooperative equilibrium*, but the stability of the equilibrium is continuously challenged by the random invasion of a small population of deceiving agents.

**Fig 6:**
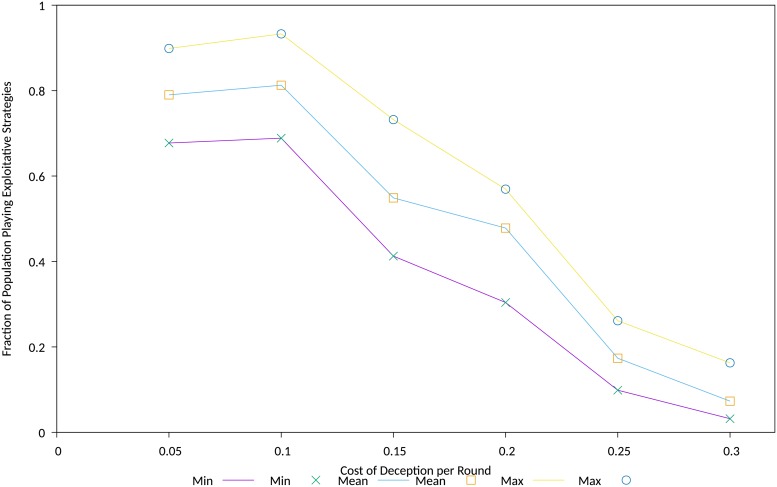
Cost dependency of deceiver population size. Fraction of population deceiving and playing exploitative IPD strategies versus the cost of deception, between 2000 and 5000 generations.

The results show in a convincing manner that agents using *Degradation* paired with the *Always Defect* strategy will successfully invade the population, and remain in the population long term, even through deceiving agents using *Degradation* struggle. The population size of such agents progressively declines, due to competition with agents that play the exploitative *Always Defect* strategy without deception, as the latter do not incur the cost of the deception. The small population of deceiving players is nevertheless able to disrupt the cooperative players, significantly reducing their numbers in the population, depicted in Fig 6.

These results are significant in two ways. The first is that even a very small population of exploitative deceivers that inject uncertainty in decision-making into a large population produce a major advantage for all players of exploitative strategies, that dominate the population while the cost of deception is low. The second is that deceiving exploitative agents suffer a significant disadvantage in fitness against non-deceiving exploitative agents who incur no costs.

Put simply, even very small and transient populations of exploitative deceiving agents drive cooperating agents out of the population, allowing them to be displaced by agents playing exploitative strategies, while damaging the overall fitness of the population by reducing the fraction of cooperating agents. This result has implications beyond the “fake news” problem, and is discussed later in this paper.

This experiment also included a Cost of *C* = 0 where deceiving agents do not suffer a disadvantage against non-deceiving agents. In this unique situation, plotted in Figs A and B in S3 Appendix, the deceiving agents playing the exploitative *All Defect* strategy invade the population and displace most strategies, other than non-deceiving *All Defect* in just over 2600 generations, producing a *collapse to a non-cooperative equilibrium*.

**Fig 7:**
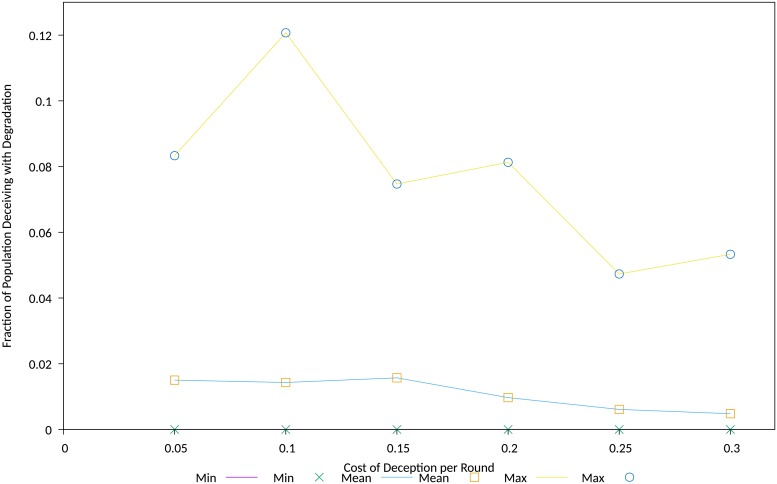
Cost dependency of exploitative population size. Fraction of population playing exploitative IPD strategies versus the cost of deception, between 2000 and 5000 generations.

This experiment also displayed pronounced dependency of the size of the population exploiting the effects of the deception on the the cost of deception, depicted in Fig 7.

### Simulation results for experiments on corruption

Figs A-I in S4 Appendix show that the diffusion behaviour closely resembles the behaviour observed with differential epidemiological models such as SIR. The fit was remarkably good. This was determined using the differential model, where integrating *E* obtained compartment estimates that were conserved to 4 decimal places, a measure of the quality of the model, and behaviour that was very similar to the simulation, including decay transients. The general behaviour observed accurately emulates initial diffusion, followed by a decay transient, in repeated patterns as observed in social media, with each cycle of diffusion and decay corresponding to the release and propagation of a popular item of “fake news”.

**Fig 8:**
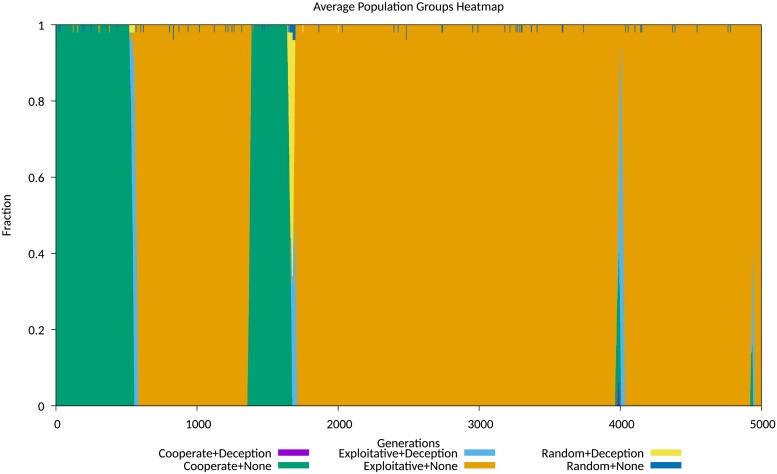
Comparison of transient and persistent population behaviours simulation run 101. Population map showing transient and persistent behaviours for simulation run 101.

**Fig 9:**
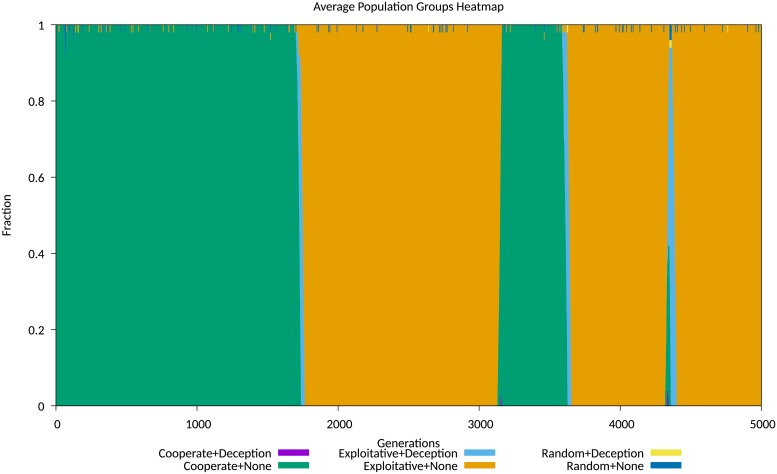
Comparison of transient and persistent population behaviours simulation run 148. Population map showing transient and persistent behaviours for simulation run 148.

No differently than in the previous experiment, the short duration effect of a deception is to produce a persistent impact on the population ratios. Agents playing exploitative strategies dominate the population for many generations following the transient presence of the deception, until cooperative strategies recover due to higher average fitness and displace the exploitative strategies. This shows the disruptive effect of a popular deception as observed empirically in social media debates. At some point the population of agents using the *Corruption* deception reappears, and the cycle is repeated again, as shown in Figs 8 and 9.

Similar behaviour was observed by Számadó et al in modelling deceptive messaging in a game of indirect reciprocity with cooperation, defection and punishment [72].

**Fig 10:**
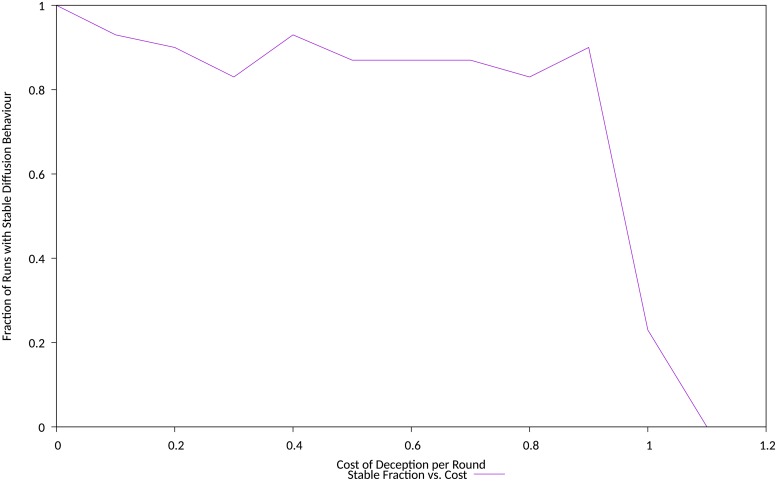
Stability of diffusion behaviour versus the cost of deception. Fraction of population exhibiting stable diffusion behaviour versus the cost of deception.

While the dependency on cost was pronounced as in the previous experiment, observed behaviour showed a weak initial cost dependency, followed by an abrupt and stronger dependency above a cost threshold, that for this simulation and its parameters occured at a cost of around 0.9, refer Fig 10. At higher costs, the population using the *Corruption* deception cannot gain a foothold in the population, and behavior is dominated by the conventional contest between populations playing cooperative and exploitative strategies.

For comparison with the experiment exploring the *Degradation* deception, we include evolving average population data for this experiment, displayed in Figs A-L in S5 Appendix. The equilibrium behaviours observed again mostly fit the *stable polymorphism* identified by Le and Boyd, with an initial population of non-deceiving agents playing cooperative strategies invaded by a mix of deceiving and non-deceiving exploitative agents, with a very similar stable but noisy equilibrium becoming established between 500 and 2,000 generations.

An interesting comparison is the effectiveness of *Corruption*, that deterministically changes the victim belief, against *Degradation*, that randomly alters victim belief. The evolving average population data shows that *Corruption* produces larger populations of exploitative agents over a much wider range of costs. This behaviour reflects empirically observed effects in propaganda and the preference for *Corruption* over *Degradation* in propaganda predating the digital age [37, 38].

Notably, this experiment included a Cost of *C* = 0 where deceiving agents do not suffer a disadvantage against non-deceiving agents. In this unique situation, plotted in Fig A in S5 Appendix, the deceiving agents playing the exploitative *All Defect* strategy invade the population and displace most strategies, other than non-deceiving *All Defect* in just over 500 generations, producing a *collapse to a non-cooperative equilibrium*.

## Discussion

The primary aim of the experiments was to demonstrate that information-theoretic models of deception were useful for more than simple taxonomical analysis of deceptions, and to determine whether an IPD simulation using agents equipped to produce deception effects could be usefully employed as a tool for modelling the effects of “fake news” in social systems such as social media. A secondary aim was to assess the effect of the cost of deceptions.

Analysis of simulation results showed remarkably good agreement with empirically observed behaviours in social media, despite a number of simplifying assumptions employed in the design of the simulation and the experiments. The ability of the simulation to capture accurately both transient short term and persistent behaviours observed empirically in social media was not expected, as we assumed that simplifications in the model could cause the simulations to depart from empirical observations of real world systems.

To the knowledge of the authors, the cost of deploying deceptions in social media has never been studied in any detail. The results showed strong cost dependencies for both experiments, albeit different in form.

Importantly, the cost to agents of performing either *Degradation* or *Corruption* deceptions strongly determined persistence of populations benefiting from deception, or whether deception was able to even establish itself in the population.

Notably, in both experiments we observed the evolution of both types of behaviours without priming the simulation with an initial population exhibiting *Degradation* and *Corruption* deceptions, demonstrating that such behaviours will evolve, invade and expand in a population where conditions permit. We also observed that exploitative strategies benefited from deceiving players without incurring costs, reflecting observed behaviours in social media.

What the simulation shows has practical implications, as increasing the cost of social media deceptions to deceiving players will reduce their ability to disrupt the population.

The cost of deception in a social system can be increased directly, by introducing penalties for deceptions, or by inoculating the population against deceptions, forcing a deceiver to employ more elaborate and expensive deceptions to achieve actual effects [80].

As yet, we have not modelled a situation where the increasing effectiveness of a deception incurs an increasing or proportionate cost. Empirical observations show that mostly more sophisticated deceptions require more effort and thus cost to execute, given some cognitive capability in the victim to recognise a deception. The relationship between deception and the cost to execute it is an empirical problem, as is the problem of how a victim might unmask a deception. This problem more generally represents the well established problem of evolutionary arms races in deceptions and means of detecting these observed in biology [39].

The results showing the sensitivity of deception effect in a population on the cost of the deception have implications beyond the immediately studied problem of “fake news” in social media. The impact of deceptions on group behaviour, especially cooperation, will be a consideration in the study of other social systems, and in evolutionary psychology.

*Shame* has been studied in IPD modelling as a mechanism to induce or maintain cooperation in a social system, Declerck et al found that exploitative players cooperated when they could not hide their greed [81]. Shaming exploitative players is a form of extrinsic cost imposed upon an exploitative player to modify their behaviour. *Degradation* or *Corruption* deceptions would permit such players to hide their greed and thus continue to play selfishly.

The agents we employed were not able to recognise failed attempts at deception, and punish the deceiving agent by defecting. The agents were also unable to associate identities with specific opponents, denying the means of identifying *a priori* deceiving players. Many strategies incorporating *Retribution* have been well studied in competitive IPD tournaments. The obvious drawback of *Retribution* alone as a mechanism to discourage deceptions is that it does not punish successful deceivers, and thus cannot achieve the effect in facilitating cooperation recently found by Kurokawa [82].

The problem of exactly how to best impose a cost on deceivers who disrupt cooperation is essentially empirical, as the specific context will determine what means are feasible.

Sewell argues from the works of Dawkins and Trivers, that implicitly selfish players will cooperate due to *reciprocal altruism*, and that displays of emotion can enable cooperation [83–85]. There is ample empirical work in the humanities showing how emotion can also be employed to support deceptions. Possibly more interesting is the manner in which deceptions could be employed to faciliate *reciprocal altruism* and thus cooperation.

The results of our experiments also show consistency with some earlier work in biology. This should not be surprising, given the generality of the information-theoretic models of deception.

Spence’s seminal work, studying job market behaviours, laid the foundations of what is termed honest signalling theory, in which signalling is represented as a game in which the signaller and receiver incur costs from honest or dishonest signalling [86]. In Spence’s model, the cost incurred to signal is employed by receivers as a measure of signaller fitness. Players incurring a higher signalling cost are thus disadvantaged, assuming the signalling accurately reflects cost.

Biology researchers have studied the related problem of fitness signalling due to its importance in mate selection, with ongoing research following Zahavi’s initial work on the “handicap principle”, whereby costly signals are employed to message fitness [87]. Grafen remapped Zahavi’s model into a game theoretic representation, and incorporated Spence’s notion of a disadvantage incurred by higher signalling cost [88, 89]. Considerable research effort has been invested since then to determine the exact relationship between honest and deceptive signalling, and cost of either [89–94].

A recurring theme in these arguments is the effect of cost upon deceptive signalling. Számadó argued that “the honesty of communication is maintained by the potential cost of cheating …” [93]. Higham argued that “… there must be a cost associated with cheating that outweighs its benefits.” In our model costs are implicit in deceptive signalling, as effort must be expended in producing the deceptive signal, in which regard it is identical to non-deceptive signalling.

The information-theoretic modelling approach differs from models frequently used in biology as it implicitly assumes a deception is imperfect, as it may or may not be successful in changing the state of belief in the victim. Therefore, other than the special case of zero cost, any cost incurred by a deception will impact the fitness of the deceiver, with severity determined by how frequently the deception fails, and the ratio of payoffs for successful versus unsuccessful deceptions. Extant research in biology is focussed mostly on the question of payoffs, the information-theoretic modelling approach indicates that the ability to better unmask deceptions will also act as a disincentive to cheating, as we know from observation of social systems.

Czárán and Hoekstra simulated the quorum sensing problem in populations of bacteria, where the organisms can cooperate or cheat, and costs were parametrised, with eight behavioural strategies present in the population [92]. Their simulations showed frequently very similar *stable polymorphism* behaviour to that observed in our experiments, with the population being invaded by deceiving agents, and cost dependent stable equilibria being attained between populations of cooperating and exploitative agents with different strategies.

Agents with the *Liar* genotype employ the *Corruption* deception, incurring a higher metabolic cost than *Ignorant* agents, that are also exploitative and unable to cooperate. Notably, Czárán and Hoekstra found similar equilibirum and non-equilibrium behaviours between *Liar* and *Ignorant* populations, as we found between deceiving and non-deceiving agents playing the *Always Defect* strategy. In both studies the lower cost incurred by the non-deceiving exploiters resulted in the latter mostly displacing the former in the population.

Clearly, many opportunities exist to apply the information-theoretic deception modelling framework and simulation methodology we describe in other problem areas, and previous work in the biology domain supports this proposition.

## Conclusion

The simulation results show, as observed empirically in social systems subjected to “fake news” attacks, that even a very small population of deceivers that appear transiently can alter the equilibrium of the population in favour of exploitative strategies, at the expense of cooperative strategies. The results also show that the ability of a population of deceivers to establish itself or remain present in a population is highly sensitive to the cost of the deception, as this cost reduces the fitness of deceivers when competing against non-deceiving agents. The observed fit of a differential model of the form of the SIR epidemiological model against diffusion behaviours observed for agents exploiting the *Corruption* deception are very close to empirically observed behaviours in social media, when fitted to such epidemiological models.

We have therefore demonstrated, using an improved formulation of the information-theoretic models of deception, that agent based evolutionary simulations employing the IPD can accurately capture the behaviours of a population subject to deception attacks using the *Degradation* deception, that introduces uncertainty, and the *Corruption* deception, that introduces a false perception. We have also demonstrated that the deceiving population diffuses into the larger population in a realistic manner.

We modelled only basic forms of the *Degradation* and *Corruption* deceptions, leaving more complex compound deceptions, including the common combination of *Corruption* and *Subversion* unexplored. The recently proposed model of inoculating the population against deceptions was also not explored. These present opportunities for future research in this area.

## Appendix

### Applicable information theory concepts and an improved model of corruption

Shannon’s model of information, and the transmission of information through a channel, is a well proven representation, ubiquitous in modelling data transmission and storage, human cognitive loads, as well as other information-centric problems [95, 96].

**Fig 11:**
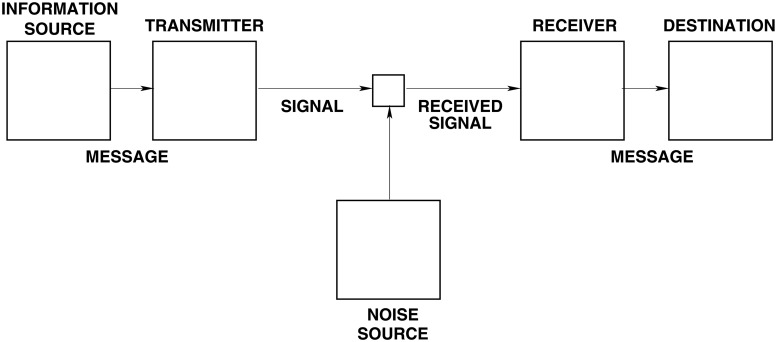
Shannon’s model for a communication channel. Shannon’s model for a communication channel, comprising a source, transmitter, channel with noise source, receiver, and destination (Kopp, per Shannon, 1948).

The Shannon model is constructed around the assumption of two entities communicating symbols through a channel, the function of which is impaired by the effects of additive white Gaussian noise (refer Fig 11). The symbols form an alphabet, which is assumed to be known and understood by all entities using the channel. The effect of additive noise in the channel is to change some symbols, thus introducing errors in transmission through the channel.

The Shannon model makes two assumptions, which are important for the study of deception. The first and weaker constraint in practice, is that noise in the channel is assumed to be Gaussian. The more important assumption is that the symbols forming an alphabet are understood by both entities, although the model makes no assumptions about the meaning of the messages encoded by the alphabet. The model assumes both entities have prior probability distributions for messages sent and received. We will show that deceptions may often involve manipulation of the channel, or misrepresentation of the meaning of messages, the latter involving manipulation of the alphabet.

Shannon’s model is quantitative, and centred on the idea of entropy. If a message contains information, an entity receiving it and understanding it will experience a state change which alters its level of uncertainty. The less likely the message, the greater its information content, noting that the prior probabilities may be unique to each entity in the channel. In particular:

$$\begin{array}{c} I\left(m\right)\;=\;-\log_{2}p\left(m\right) \end{array}$$

where *I*(*m*) is the information in message *m*, and *p*(*m*) is the probability of the message.

In the context of deception, Shannon’s channel capacity theorem is much more useful. It states that the capacity of a channel to carry information depends on the magnitude of the signal encoding the symbols, the magnitude of the interfering noise in the channel, and the bandwidth of the channel:

$$\begin{array}{c} C=W\log_{2}(1+\frac{S}{N}) \end{array}$$

The channel capacity theorem is defined in its basic form for a physical channel, with the properties of capacity *C* in bits/s, bandwidth *W* in Hertz or cycles/s, and signal power *S* and noise power *N* in Watts, reflecting the definition of the theorem for a physical communication channel. Actions by a transmitting entity to manipulate the terms in the capacity equation will, in turn, manipulate the internal state of the receiving entity.

The application of models of deception based on the channel capacity theorem is now well established in areas involving electrical transmission or detection of data [46]. This is because the channel capacity theorem can be easily and directly mapped into widely used models in this area.

In other systems, additional mappings to transform variables into a form suitable for the capacity theorem model are required. An example might be a victim flooded with a large number of text documents, mostly containing repetitive or irrelevant content, intended to reduce the “signal to noise ratio” seen by the victim. An applicable and context sensitive mapping into Shannon’s *S*/*N* ratio must be performed to produce a quantitative measure, although this may not be required if the intent is simply to understand how the deception alters a game, and perform ordinal ranking of outcome preferences.

Another important concept in information theory for understanding deception is that of measures of similarity or difference, since similarity to or differences from a known message may be used as a means of distinguishing valid from invalid messages, or as a means of deception, making a false message appear to be real. This is the basis for the model employed to describe *Corruption* deceptions.

Measures of similarity or difference remain an area of active research in information theory, and at this time a number of measures have been proposed, with varying degrees of generality. For instance, Vitanyi and Li et al have proposed measures based on information distance, while Lin proposed a similarity theorem that defines a measure of similarity by the ratios of information [97–99].

Vitanyi’s measure of difference is presented as one example. How different *Y* is from *X* is measured by the size *K*(*Y*|*X*) of the description of how to edit *Y* to turn it into *X*. If *Y* is the same as *X*, then there is no editing and the difference is 0. Since the edits required to turn *Y* into *X* might be different from those required to turn *X* into *Y*, the maximum is taken. However, this is divided by the maximum size of the edits *K*(*X*) and *K*(*Y*) required to construct *X* and *Y* from scratch. So, difference is taken in proportion to the complexity of *X* and *Y* alone. The precise formula used by Vitanyi is as follows:

$$\begin{array}{c} D\left(X,Y\right)=\frac{K(XY)-min(K(X),K(Y))}{max(K(X),K(Y))}\\ S(X,Y)=\;1\;-\;D(X,Y) \end{array}$$

Where *S* is similarity, *D* is difference, and *K* is the editing function applied to *X*, *Y*. Vitanyi uses a theoretically optimal editing process. But, for practical purposes he suggests the use of any common compression algorithm, such as ZIP. The typical compression algorithm is dictionary based. It stores common words and phrases from *X* and writes the list of code numbers. If *Y* contains few words or phrases that do not already exist in *X*, then the compression of *XY* will be only slightly larger than of *X*. Such an optimal process may or may not be realised in practice, when a player attempts to determine similarity.

A number of other widely employed measures of similarity or difference exist and could be applied here. For instance Kullback-Leibler divergence or relative entropy, or its second derivative, the Fisher information metric, are often employed to gauge differences between two probability distributions [100, 101].

Kullback-Leibler divergence is:

$$\begin{array}{c} D_{KL}\left(P||Q\right)=\sum_{X}P\left(X\right)log_{2}\left(\frac{P(X)}{Q(X)}\right) \end{array}$$

where *D*_*KL*_(*P*||*Q*) is the KL divergence in bits, and *P*(*X*) and *Q*(*X*) are some distributions of random variable *X*. If the distributions are not identical, divergence is non-zero:

$$\begin{array}{c} D_{KL}\left(P||Q\right)\;\neq \;0\Leftrightarrow P\left(X\right)\;\neq \;Q\left(X\right) \end{array}$$

KL divergence is not symmetrical, but still provides a useful measure of difference. Mutual information is also a useful means of establishing differences, and is of interest given its implicit relationship with Shannon information. A common definition is [102]:

$$\begin{array}{c} I\left(X,Y\right)=\sum_{X}\sum_{Y}P\left(X,Y\right)log_{2}\left(\frac{P(X,Y)}{P(X)P(Y)}\right) \end{array}$$

Where *P*(*X*, *Y*) is the joint distribution, and *P*(*X*) and *P*(*Y*) are the respective distributions for *X*, *Y*.

The empirical problem in modelling deceptions will often lie in determining how an entity measures similarity, and which measure or metric best captures or approximates this behaviour.

Shannon’s channel capacity, and information-theoretic measures of similarity are bounds, respectively, on systems which transmit or compare messages. An actual non-ideal physical system may not be capable of achieving these bounds, and may have a decision threshold well below these bounds. As a result, a valid message may be lost in noise internal to the system, or two similar messages might be interpreted to be identical, despite being very different in some way.

Deceptions exploit these limitations of non-ideal systems. Put quantitatively:

$$\begin{array}{c} C_{receiver}\;\leq \;C_{objective}\\ S_{receiver}\left(A,B\right)\;\neq \;S_{objective}\left(A,B\right) \end{array}$$

Where *C*_*receiver*_ is the channel capacity actually available to the victim, *C*_*objective*_ the achievable channel capacity, *S*_*receiver*_(*A*, *B*) the similarity between *A* and *B* as perceived by the receiver, while *S*_*objective*_(*A*, *B*) is the objective similarity, assuming an actual objective or “ground truth” rather than subjective probability distribution. Successful deceptions arise when the deceiver manipulates the channel or message in a manner, which the receiver is unable recognise or overcome, altering the receiver’s perception, and thus subjective probabilities. For simplicity, we further assume that the outcome of a deception is a discrete state change in the receiver’s perception, a reasonable assumption since in practice a successful deception typically captures the receiver.

## Supporting information

S1 Appendix Simulation parameters and outputs, table of experiments, table of abbreviations, simulator validation experiments. Supporting materials.
(PDF)

S2 Appendix Iterated prisoner’s dilemma with deception, examples. A more detailed description of the IPD model, deception model integration, with a number of examples.
(PDF)

S3 Appendix Cost dependency of populations in degradation experiments. Odd numbered plots show the observed population sizes for agents grouped by IPD strategy played, and whether they employ the *Degradation* deception, or not, parametrised by cost. Even numbered plots show the fractions of the agent population playing respective IPD strategies. Tabulated data shows statistical data for equilibrium behaviour of the *stable polymorphism*.
(PDF)

S4 Appendix Cost dependency of differential model fit in corruption experiments. These plots show the observed transient diffusion behaviour for exploitative strategies, comprising agents employing *Always Defect*, with or without the concurrent use of the *Corruption* deception, parametrised by cost.
(PDF)

S5 Appendix Cost dependency of populations in corruption experiments. Depicted plots show the observed population sizes for agents grouped by IPD strategy played, parametrised by cost. Tabulated data shows statistical data for equilibrium behaviour of the *stable polymorphism*.
(PDF)

S1 Data Tabulated data for S3 Appendix. (CSV)

S2 Data Tabulated data for S5 Appendix. (CSV)

S3 Data Tabulated data for Fig 6. (CSV)

S4 Data Tabulated data for Fig 7. (CSV)
