# Supplementary material for: Information-theoretic models of deception: Modelling cooperation and diffusion in populations exposed to "fake news"
Source: PLoS One. 2018 Nov 28;13(11):e0207383. doi: 10.1371/journal.pone.0207383 (PMC6261583; doi:10.1371/journal.pone.0207383)
Supplement: S1 Appendix — Supporting materials. (PDF) [file pone.0207383.s001.pdf]

S1 Appendix

Simulation Parameters and Outputs

This appendix section summarises simulation parameters and variables of interest.

Heritable Agent Traits

The agents all possess heritable traits that determine their behaviour.

- `mutation-prob` - the probability that mutation will alter agent traits during reproduction
- `IW-method` - which of the four deception models the agent will exclusively use, or None
- `IPD-strategy` - one of a number of hardcoded IPD game strategies that the agent will use

Input Parameters and Outputs

These were the parameters for the simulation:

- `generations` - 5000 was employed
- `population-size` - 50 was employed due to computational time constraints
- `iterations` - 100 was employed
- `IW-costs-string` - relative costs of deception attacks
- `IPD-permitted` - controls experiment mode
- `IW-permitted` - controls experiment mode
- `Initial-Population` controls experiment mode

The experiments were both run with only one deception model permitted, although the simulation was designed to permit a mix of deception models employed, each with unique cost per deception. Deception cost was incremented in steps of 0.1 across multiple simulation runs.

Simulation Outputs

- Seed value for the random number generator
- total agent score mean and standard deviation
- average per game score mean and standard deviation
- number of agents using Degradation
- number of agents using Corruption

- number of agents using Denial
- number of agents using Subversion
- number of agents using no deception model
- number of agents playing Tit for Tat
- number of agents playing Tit for 2 Tats
- number of agents playing Pavlov
- number of agents playing Always Defect
- number of agents playing Always Cooperate
- number of agents playing Random
- number of agents playing Probabilistic
- gain from deception attacks mean and standard deviation
- gain from deception attacks for Degradation mean and standard deviation
- gain from deception attacks for Corruption mean and standard deviation
- gain from deception attacks for Denial mean and standard deviation
- gain from deception attacks for Subversion mean and standard deviation
- success probability of deception attacks for Degradation mean and standard deviation
- success probability of deception attacks for Corruption mean and standard deviation
- success probability of deception attacks for Denial mean and standard deviation
- success probability of deception attacks for Subversion mean and standard deviation
- success probability of deception attacks for None mean and standard deviation
- effects of deception attacks for all agents
- effects of deception attacks for Degradation
- effects of deception attacks for Corruption
- effects of deception attacks for Denial
- effects of deception attacks for Subversion
- unknown-history-response-prob mean and standard deviation
- mutation-prob mean and standard deviation

Table of Experiments

Deception Type	Cost	Initial Population Mix	ID	Runs	Stability Behaviour
Degradation	0	TFT, TF2T, RND, PVL, PRB, AC	Yes	30	Collapse to NC Equilibrium
Degradation	0.05	TFT, TF2T, RND, PVL, PRB, AC	Yes	30	Stable Polymorphism
Degradation	0.1	TFT, TF2T, RND, PVL, PRB, AC	Yes	30	Stable Polymorphism
Degradation	0.15	TFT, TF2T, RND, PVL, PRB, AC	Yes	30	Stable Polymorphism
Degradation	0.2	TFT, TF2T, RND, PVL, PRB, AC	Yes	30	Stable Polymorphism
Degradation	0.25	TFT, TF2T, RND, PVL, PRB, AC	Yes	30	Stable Polymorphism
Degradation	0.3	TFT, TF2T, RND, PVL, PRB, AC	Yes	30	Stable Polymorphism
Degradation	0.05	TFT, TF2T, RND, PVL, PRB, AC	No	30	Stable Polymorphism
Degradation	0.1	TFT, TF2T, RND, PVL, PRB, AC	No	30	Stable Polymorphism
Degradation	0.15	TFT, TF2T, RND, PVL, PRB, AC	No	30	Stable Polymorphism
Degradation	0.2	TFT, TF2T, RND, PVL, PRB, AC	No	30	Stable Polymorphism
Degradation	0.25	TFT, TF2T, RND, PVL, PRB, AC	No	30	Stable Polymorphism
Degradation	0.3	TFT, TF2T, RND, PVL, PRB, AC	No	30	Stable Polymorphism
Corruption	0	TFT, TF2T, RND, PVL, PRB, AC	No	30	Collapse to NC Equilibrium
Corruption	0.1	TFT, TF2T, RND, PVL, PRB, AC	No	30	Stable Polymorphism
Corruption	0.2	TFT, TF2T, RND, PVL, PRB, AC	No	30	Stable Polymorphism
Corruption	0.3	TFT, TF2T, RND, PVL, PRB, AC	No	30	Stable Polymorphism
Corruption	0.4	TFT, TF2T, RND, PVL, PRB, AC	No	30	Stable Polymorphism
Corruption	0.5	TFT, TF2T, RND, PVL, PRB, AC	No	30	Stable Polymorphism
Corruption	0.6	TFT, TF2T, RND, PVL, PRB, AC	No	30	Stable Polymorphism
Corruption	0.7	TFT, TF2T, RND, PVL, PRB, AC	No	30	Stable Polymorphism
Corruption	0.8	TFT, TF2T, RND, PVL, PRB, AC	No	30	Stable Polymorphism
Corruption	0.9	TFT, TF2T, RND, PVL, PRB, AC	No	30	Stable Polymorphism
Corruption	1	TFT, TF2T, RND, PVL, PRB, AC	No	30	Stable Polymorphism
Corruption	1.1	TFT, TF2T, RND, PVL, PRB, AC	No	30	Stable Polymorphism

Table S1. This table shows experiments conducted, excluding test and calibration runs employed to validate proper operation of the simulation tool. Abbreviations employed detailed below. Results are summarised and detailed in **S3 Appendix, S4 Appendix** and **S5 Appendix**.

Experimental Datasets

Raw and postprocessed simulation data, and plotting scripts, are available at the Monash University figshare repository: `http://dx.doi.org/10.26180/5b4d965923ca6`.

Table of Abbreviations

**AC**	Always Cooperate
**AD**	Always Defect
**C**	Cooperate
**COR**	Corruption deception
**D**	Defect
**DEG**	Degradation deception
**DEN**	Denial deception
**ID**	Initial Deceiver (population)
**IPD**	Iterated Prisoner's Dilemma
**NC**	Non-cooperative
**PRB**	Probabilistic
**PVL**	Pavlov
**RND**	Random
**SEIZ**	Bettencourt's *Susceptible Exposed Infected Skeptical* compartment model
**SIR**	*Susceptible Infectious Recovered* compartment model
**SUB**	Subversion deception
**TFT**	Tit For Tat
**TF2T**	Tit For Two Tats

Table S2. This table lists the various abbreviations employed.

Simulator Validation Experiments

These plots comprise a set of special cases, employed to validate the function and performance of the *Netlogo* simulation tool we employed.

The first case in Fig A is where there are no deceiving agents in the population, as the simulator algorithms for deception have been disabled. The purpose of this case is to show the equilibrium behaviour of the respective subpopulations employing different IPD strategies without deception.

The results are very similar to simulation cases with very high costs, despite the fact that in the high cost simulations, deceiving agents are continuously evolved and enter the population.

The second case in Fig B is for a very high cost, such that deceiving agents are unable to establish themselves in the population due to very low fitness. We employed the value of Cost $C = 5.0$ as it was more than sixteen times higher than the highest cost employed in the *Degradation* experiment. The results are consistent with the case of Cost $C = 0.3$.

The third case in Fig C is a consistency check with a larger population of 150, rather than the 50 employed in the two main experiments, as in Fig D.

The value of 150 was chosen as it is Dunbar's number for a social system. A Cost $C = 0.15$ was employed as it shows a pronounced effect of *Degradation* in a population of 50 agents. The effect of tripling the population size is improved stability in populations, as the effect of invading deceivers is damped down, and a slightly increased sensitivity to Cost, with populations ratios much closer to those observed at a Cost $C = 0.2$.

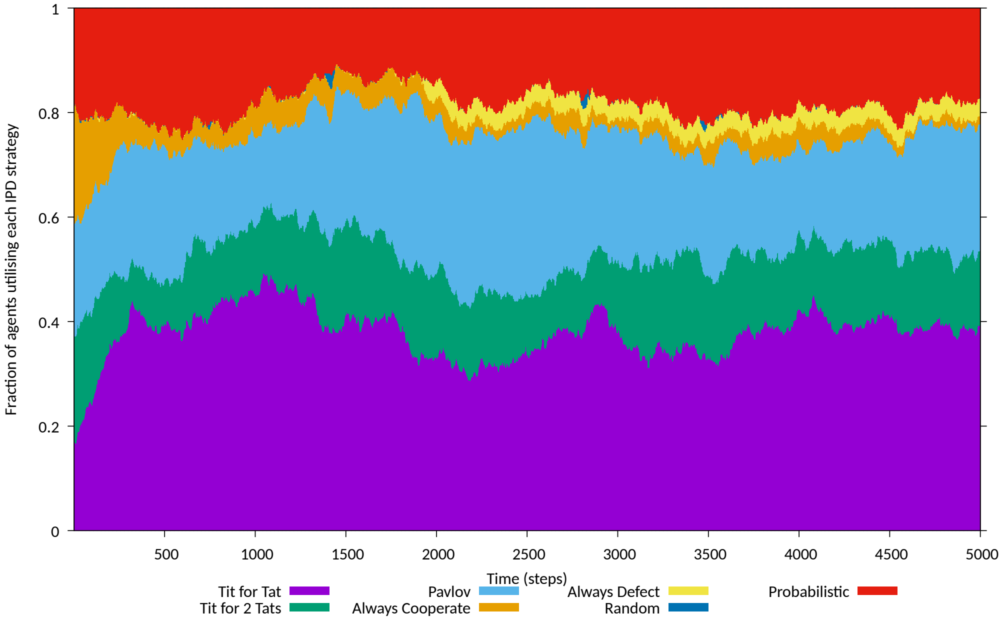

Fig. A. IPD Strategy Subpopulations for simulation with all deceptions disabled for $T = 5$, $R = 3$, $P = 1$, $S = 0$.

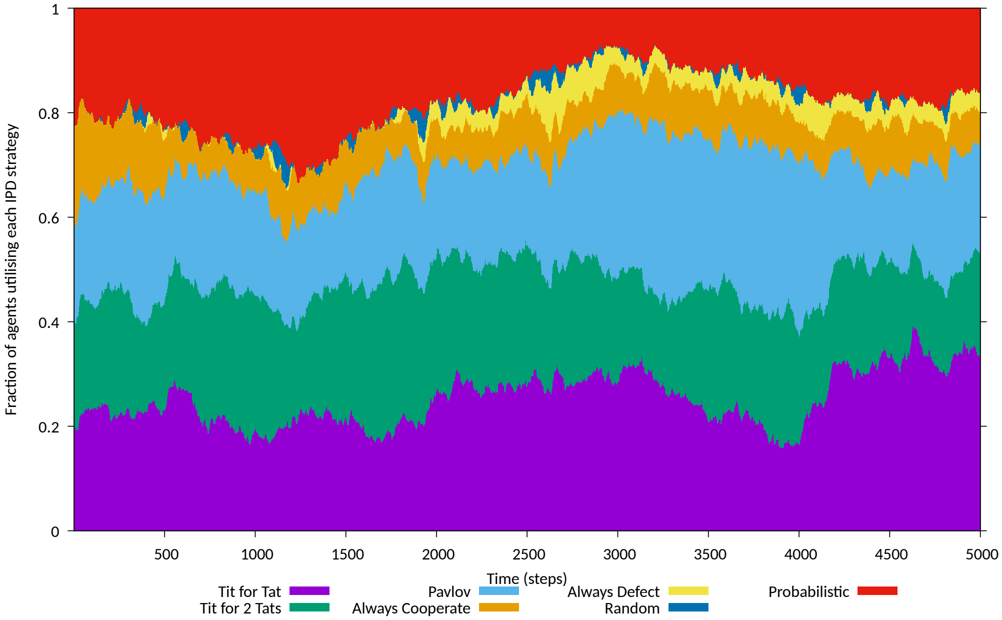

Fig. B. IPD Strategy Subpopulations for Cost $C = 5.0$, $T = 5$, $R = 3$, $P = 1$, $S = 0$.

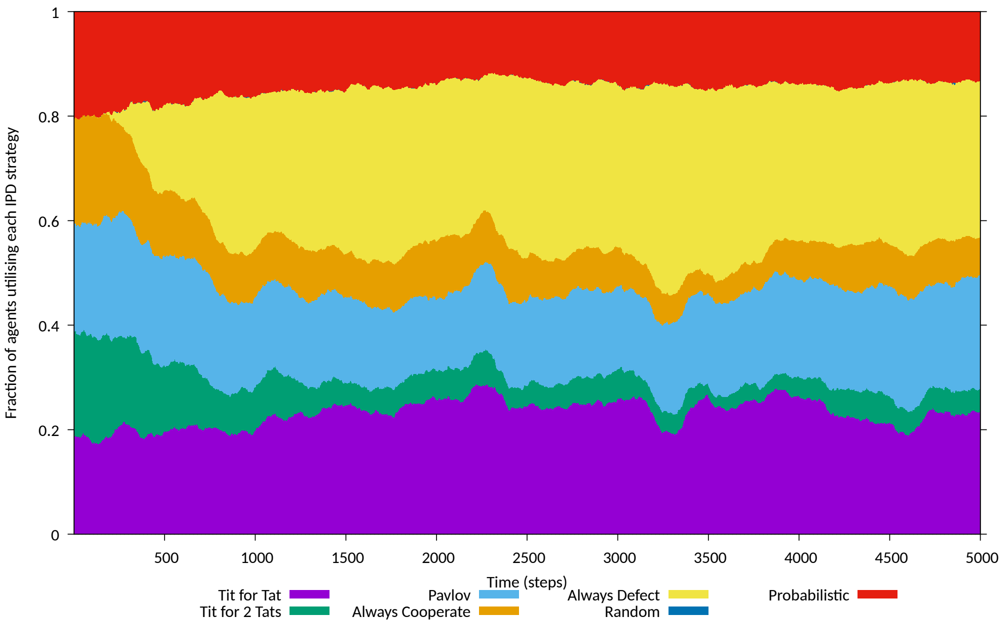

Fig. C. IPD Strategy Subpopulations for Cost $C = 0.15$, $T = 5$, $R = 3$, $P = 1$, $S = 0$, with a population size of 150 rather than 50 employed in both main experiments.

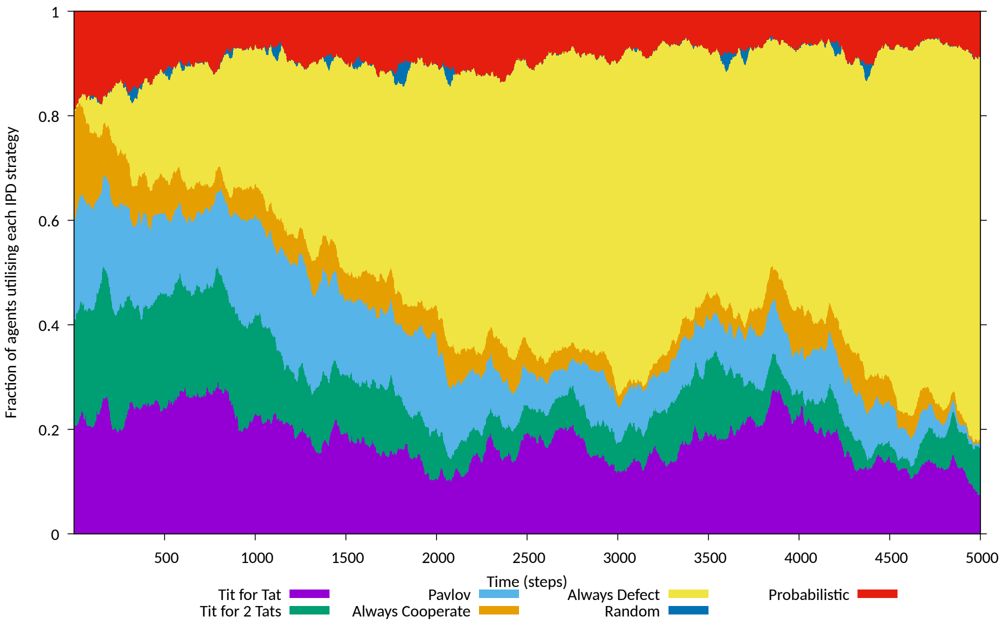

Fig. D. Degradation in Population for Cost $C = 0.15$, $T = 5$, $R = 3$, $P = 1$, $S = 0$, with a population size of 50.
