# Supplementary material for: Information-theoretic models of deception: Modelling cooperation and diffusion in populations exposed to "fake news"
Source: PLoS One. 2018 Nov 28;13(11):e0207383. doi: 10.1371/journal.pone.0207383 (PMC6261583; doi:10.1371/journal.pone.0207383)
Supplement: S2 Appendix — A more detailed description of the IPD model, deception model integration, with a number of examples. (PDF) [file pone.0207383.s002.pdf]

S2 Appendix

Iterated Prisoner's Dilemma with Deception

The model employed incorporates a mechanism for deception based on the four information theoretic models. A deception is considered successful if it alters the outcome of the round in a manner that the deceiving agent sought. It is considered unsuccessful otherwise.

For the IPD game, the deceiving agent aims to maximise its payoff. The Netlogo PD game we employed has the following parameters:

**Agent A \Agent B**	**Agent B Cooperate**	**Agent B Defect**
**Agent A Cooperate**	A: R=3<br>B: R=3	A: S=0<br>B: T=5
**Agent A Defect**	A: T=5<br>B: S=0	A: P=1<br>B: P=1

Table S1. Netlogo Prisoner's Dilemma.

Where:

- Temptation (b): $T = 5$
- Reward ($b - c$): $R = 3$
- Punishment: $P = 1$
- Sucker: $S = 0$

Each agent has a memory that records the three previous moves played by the random opponents the agent encountered previously. The four deception models operate on the memory of an opponent, overwriting it with a deceptive recollection of previous agent encounters. This models the effect of a deception that alters how a victim perceives its environment:

- **Degradation:** The last three moves of the victim's memory are replaced with random moves. This emulates the injection of noise into the channel.
- **Corruption:** *Defects* in the last three moves of the victim's memory are changed to *Cooperates*. This alters the victim agent's perception to a false belief that the previous defecting agents were cooperative.
- **Denial:** Victim agent is unable to perceive the attacker's moves for one turn and retaliates with a *Defect*.
- **Subversion:** Causes victim to *Cooperate* unconditionally for the next round, overriding the victim's IPD strategy.

The intent of an agent employing an exploitative IPD strategy and a deception is to earn a payoff of $T = 5$, leaving the victim with a payoff of $S = 0$. If the deception, which might be *Degradation*, *Corruption*, *Denial* or *Subversion*, alters the victim's memory such that the victim playing its strategy returns a payoff to the attacker of $T = 5$ and a payoff to the victim of c, the deception is considered successful.

Consider a an agent playing the cooperative *Tit-For-Tat* strategy, that has most recently encountered opponents all of whom defected. As the last opponent defected, it would defect on its next encounter if playing the unaltered game. If the opponent is a deceiving agent, the last encounter with an opponent may or may not be remembered correctly.

The *Degradation* deception will randomly flip the remembered defections, so there is a 50% probability the last defection would be falsely remembered as a cooperation, and thus a 50% probability the agent would play a *Cooperate* rather than the *Defect* it would have played. Therefore there is a 50% probability the attacking agent will earn its intended payoff of $T = 5$, and leave the victim with $S = 0$.

The *Corruption* deception will overwrite all previous *Defects* with *Cooperates*, so the agent being deceived will play its *Tit-For-Tat* strategy, and play a *Cooperate* rather than the *Defect* it would have played. The attacking agent will earn its intended payoff of $T = 5$, and leave the victim with $S = 0$.

Different deception models produce different effects, and their probability of success depends on the victim agent's strategy. For instance a victim agent playing the *Always Defect* strategy will simply ignore the deception and play *Defect*.

The matrix of possible encounters in a mixed population of deceiving and non-deceiving agents with arbitrary strategies is thus:

- *No Deception:* Player A and Player B do not deceive, unaltered IPD strategy outcomes are employed

- *Deception by Player A:* Player A is a deceiver, Player B is a non-deceiver, player A deceives and plays its IPD strategy (we record success or failure of deception), while for player B, the IPD strategy outcome is determined by the effect of the deception on its IPD strategy

- *Deception by Player B:* Player B is a deceiver, Player A is a non-deceiver, player B deceives and plays its IPD strategy (we record success or failure of deception), while for player A, the IPD strategy outcome is determined by the effect of the deception on its IPD strategy

- *Mutual Deception:* Both players A and B are deceivers. We calculate the respective payoffs for both players without deceptions and save the payoffs in a temporary variable. We apply the respective deceptions to the memories of both players, and then calculate the respective payoffs for both players with deceptions. We use the saved payoff values in the temporary variables to calculate the success or failure of the respective deceptions.

The problem of mutual deception was especially interesting. Other than the unique information theoretic *Denial* deception, where the victim knows it is being deceived and thus can respond with the *Defect*, in all of the other deceptions the victim does not know it is being deceived. The strategies of *Always Defect* and *Always Cooperate* are immune to deceptions as the strategy does not depend on agent state information, in this instance the memory of past encounters. In all other strategies, the agents will choose their play in a manner determined by the strategy played and history of past opponent moves. As in the instance of a agent deceiving a non-deceiving victim agent, the deceiving agent will choose a move based on its strategy and its history of past encounters, but that history may have been corrupted by a deception. To determine whether a deception was successful or not we have to compare the payoff against the payoff that would have been earned without a deception. In the case of a concurrent mutual deception, both agents corrupt each others' histories before they execute their play. To provide a baseline to determine whether the deception was successful, we first compute payoffs for both agents without deception, and

Agent A Last Opponent	Agent B Deception	Deception Outcome	Agent A Payoff	Agent B Payoff	Agent B Score C=1
C	DEG	Random	0 or 1	5 or 1	4 or 0
C	COR	Fail	0	5	4
C	DEN	Fail	1	1	0
C	SUB	Fail	0	5	4
D	DEG	Random	0 or 1	5 or 1	4 or 0
D	COR	Success	0	5	4
D	DEN	Fail	1	1	0
D	SUB	Success	0	5	4

Table S2. Payoffs and Score for Agent A playing *Tit-For-Tat (TFT)* against deceiving Agent B playing *Always Defect (AD)*, for a deception cost of C=1.

then compute payoffs with deception, and if the deception produced a higher payoff, record it as successful.

Each agent employs a *Score* which is a cumulative sum of payoffs, and is used as a measure of fitness in the evolutionary simulation. The global *Cost* variable is subtracted from the *Score* of every deceiving agent in every encounter. This reflects the real world constraint that producing or propagating a deceptive message requires some effort and thus cost, even if it is a small value.

Assuming a *Cost* value of $C = 1$, in the previous example where the successful deceiving agent earned a payoff of $T = 5$, the *Score* would be increased at the end of the encounter by the value of the payoff less the *Cost*, i.e. $Score_N = Score_{N-1} + 5 - 1$.

Table S2 shows payoffs and Agent B scores for the very commonly observed encounter between a non-deceiving Agent A using the *Tit-For-Tat* strategy and a deceiving Agent B using the *Always Defect* strategy, for a deception cost of $C = 1$.
