# Supplementary material for: Information-theoretic models of deception: Modelling cooperation and diffusion in populations exposed to "fake news"
Source: PLoS One. 2018 Nov 28;13(11):e0207383. doi: 10.1371/journal.pone.0207383 (PMC6261583; doi:10.1371/journal.pone.0207383)
Supplement: S3 Appendix — Odd numbered plots show the observed population sizes for agents grouped by IPD strategy played, and whether they employ the Degradation deception, or not, parametrised by cost. Even numbered plots show the fractions of the agent population playing respective IPD strategies. Tabulated data shows statistical data for equilibrium behaviour of the stable polymorphism. (PDF) [file pone.0207383.s003.pdf]

S3 Appendix

Cost Dependency of Populations in Degradation Experiments

Odd numbered plots show the observed population sizes for agents grouped by IPD strategy played, and whether they employ the Degradation deception, or not, parametrised by cost. Data for each generation are averaged over thirty simulation runs computed with identical random seeds.

Even numbered plots show the fractions of the agent population playing respective IPD strategies. Data for each generation are averaged over thirty simulation runs computed with identical random seeds..

Tabulated data shows statistical data for equilibrium behaviour of the *stable polymorphism*.

Cooperative+Deception Agents employing deception concurrently with a cooperative strategy, i.e. TFT, TF2T, Pavlov, Probabilistic, Always Cooperate

Cooperative+None Agents not employing deception concurrently with a cooperative strategy, i.e. TFT, TF2T, Pavlov, Probabilistic, Always Cooperate

Exploitative+Deception Agents employing deception concurrently with an exploitative strategy, i.e. Always Defect

Exploitative+None Agents employing deception concurrently with an exploitative strategy, i.e. Always Defect

Random+Deception Agents employing deception concurrently with the Random strategy, included as a control

Random+None Agents not employing deception concurrently with the Random strategy, included as a control

Observed behavior shows that the population of agents that employ the Degradation deception is always small, and typically transient, as even a very low cost burden makes these agents less able to compete with exploitative agents that play the Always Defect strategy.

The TFT, TF2T, Pavlov and Probabilistic strategies compete against the Always Defect strategy, with a directly observable disadvantage incurred by the latter as the cost of deceptions increases.

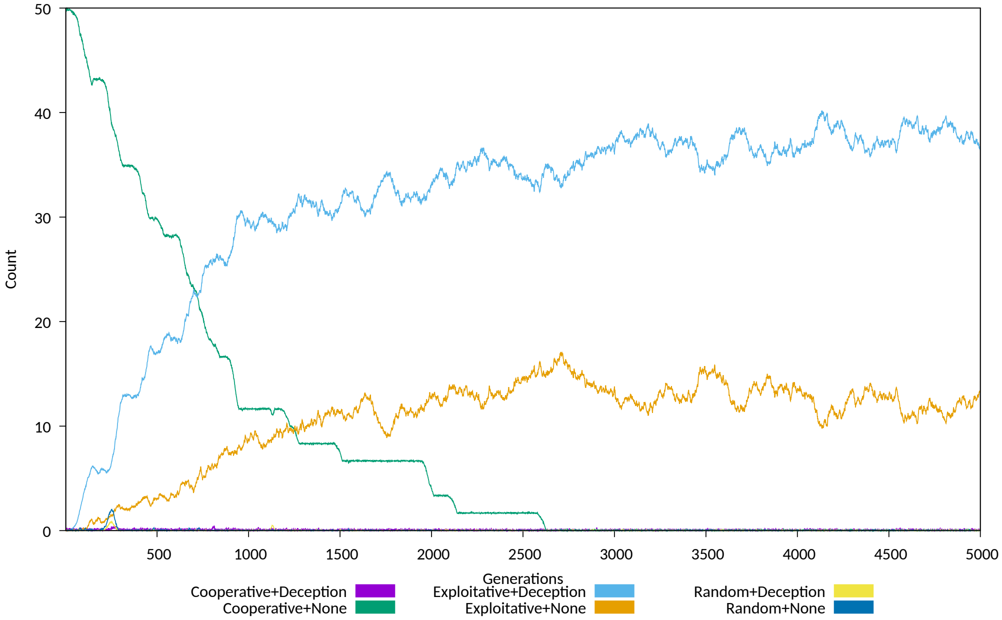

Fig. A. Degradation in Population for Cost $C = 0.0$, $T = 5$, $R = 3$, $P = 1$, $S = 0$.

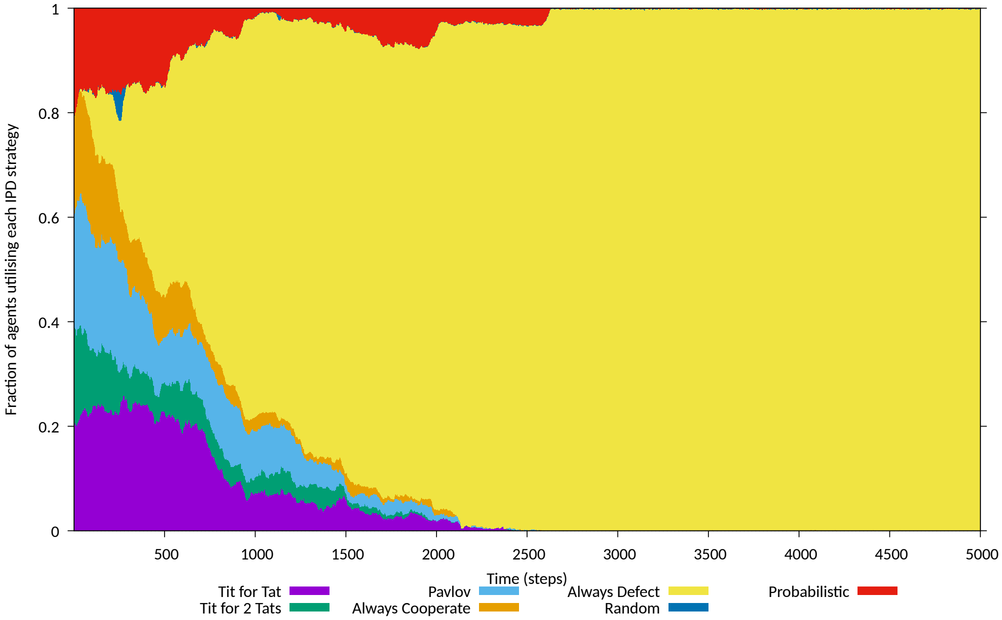

Fig. B. IPD Strategy Subpopulations for Cost $C = 0.0$, $T = 5$, $R = 3$, $P = 1$, $S = 0$.

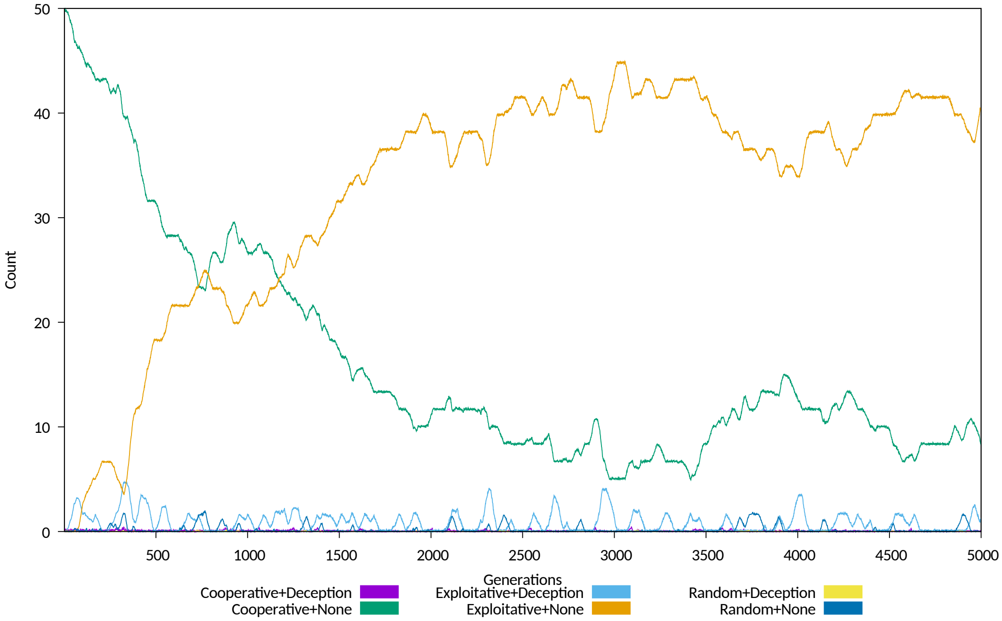

Fig. C. Degradation in Population for Cost $C = 0.05$, $T = 5$, $R = 3$, $P = 1$, $S = 0$.

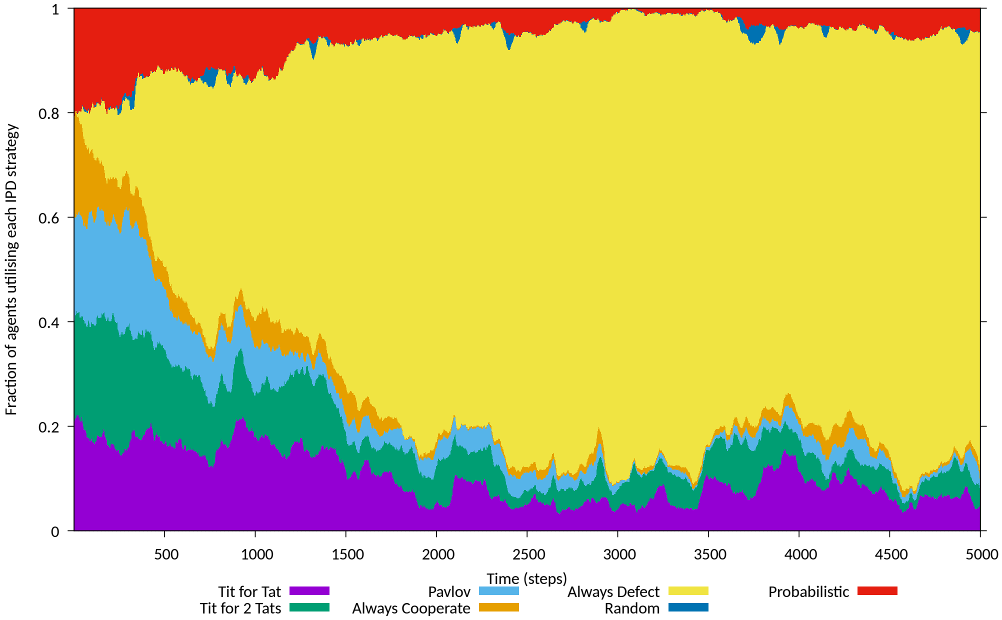

Fig. D. IPD Strategy Subpopulations for Cost $C = 0.05$, $T = 5$, $R = 3$, $P = 1$, $S = 0$.

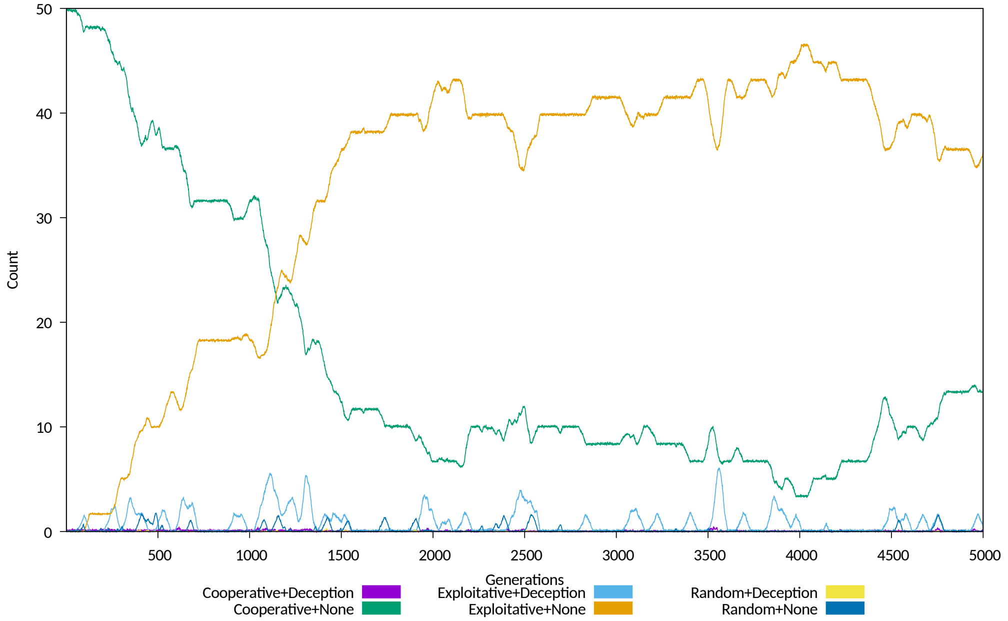

Fig. E. Degradation in Population for Cost $C = 0.1$, $T = 5$, $R = 3$, $P = 1$, $S = 0$.

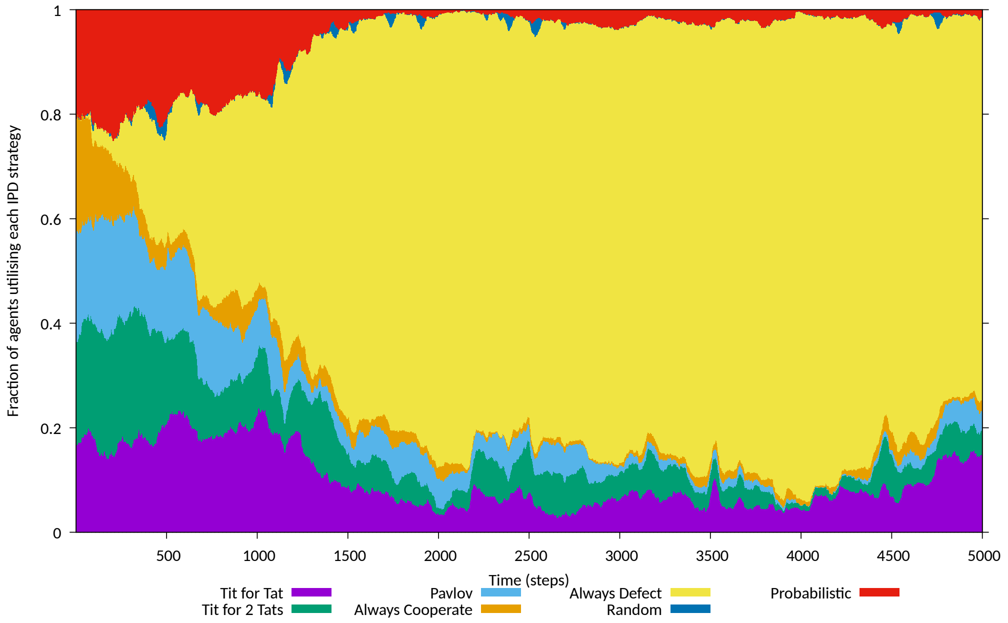

Fig. F. IPD Strategy Subpopulations for Cost $C = 0.1$, $T = 5$, $R = 3$, $P = 1$, $S = 0$.

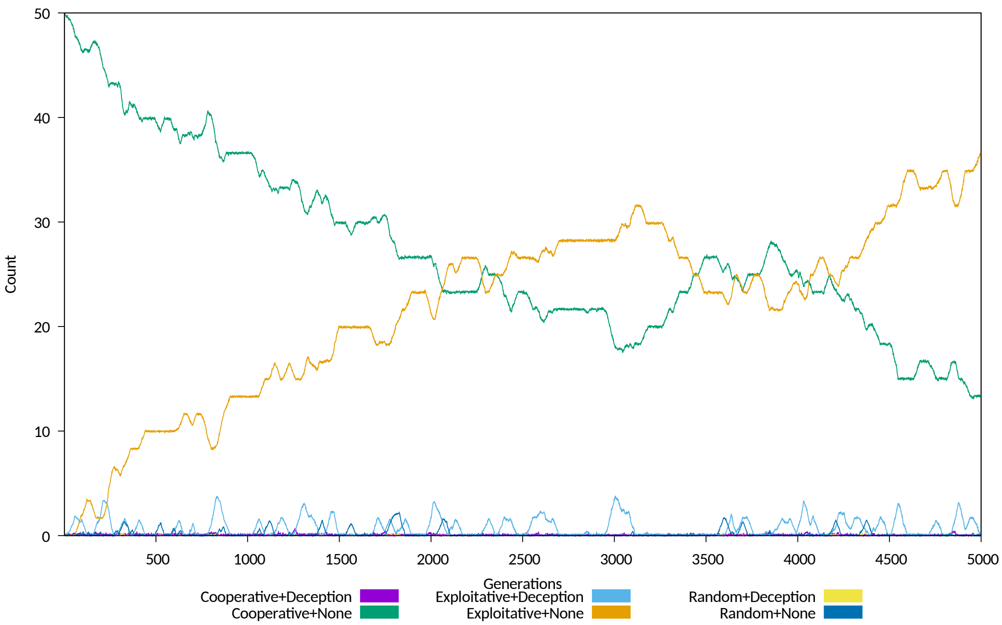

Fig. G. Degradation in Population for Cost $C = 0.15$, $T = 5$, $R = 3$, $P = 1$, $S = 0$.

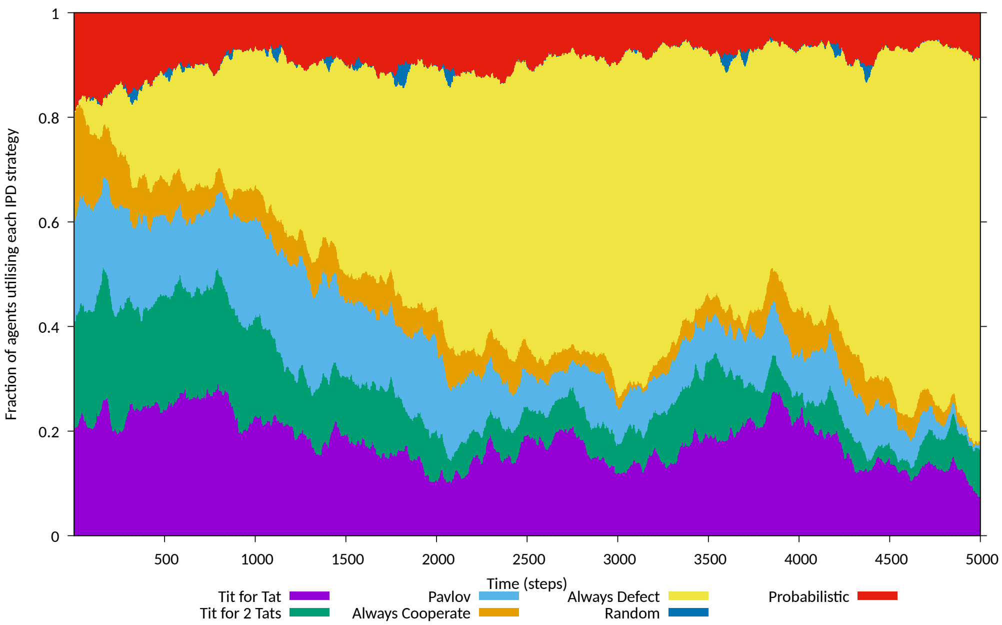

Fig. H. IPD Strategy Subpopulations for Cost $C = 0.15$, $T = 5$, $R = 3$, $P = 1$, $S = 0$.

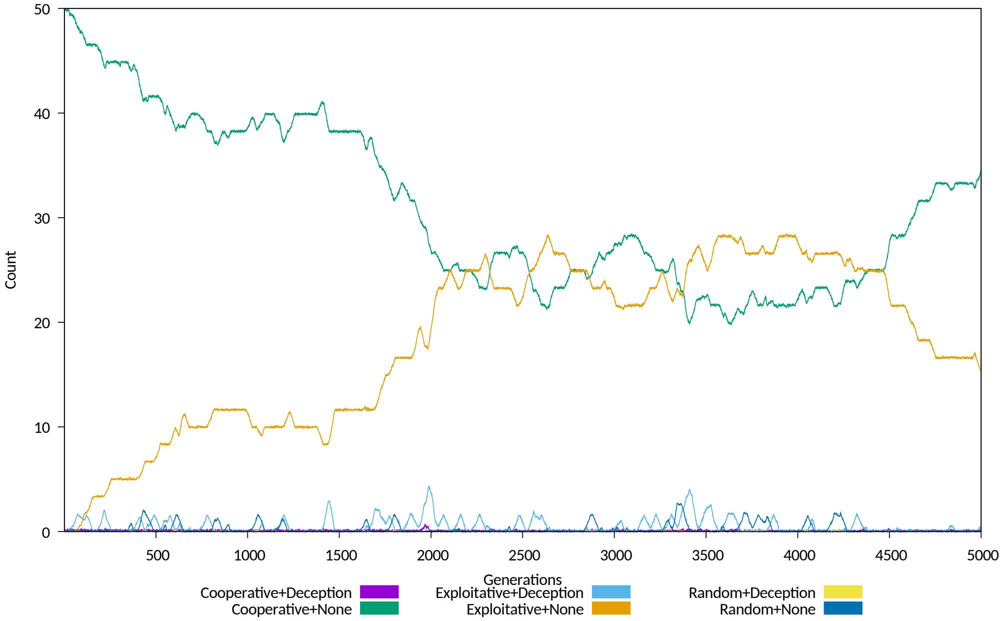

Fig. I. Degradation in Population for Cost $C = 0.2$, $T = 5$, $R = 3$, $P = 1$, $S = 0$.

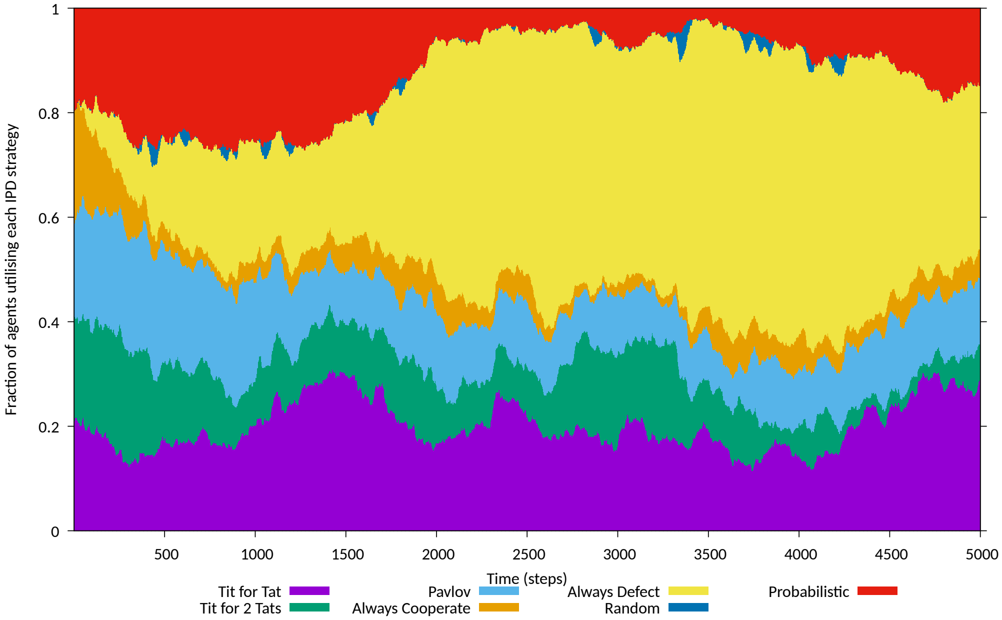

Fig. J. IPD Strategy Subpopulations for Cost $C = 0.2$, $T = 5$, $R = 3$, $P = 1$, $S = 0$.

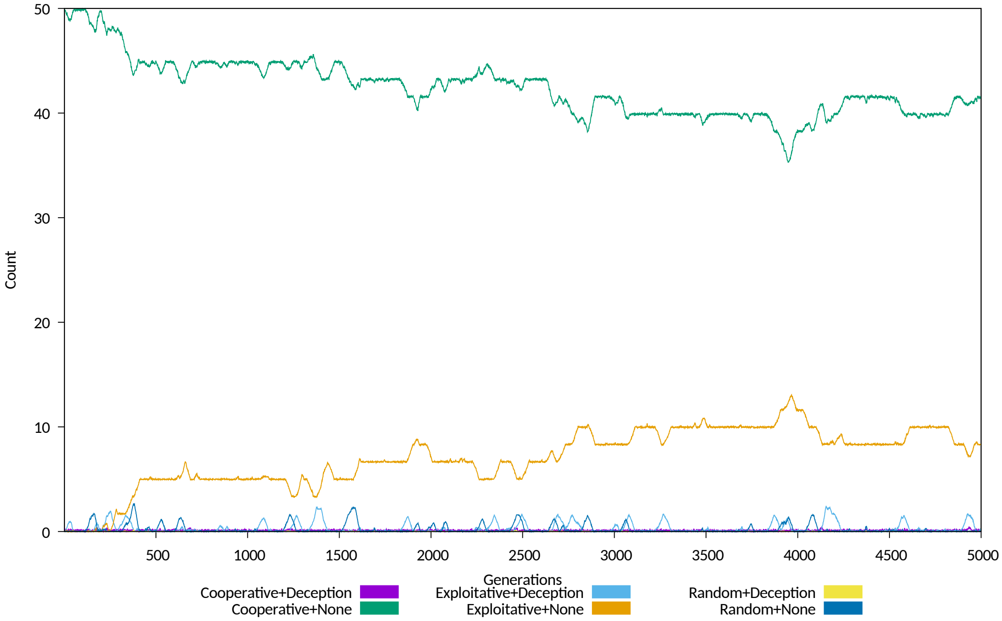

Fig. K. Degradation in Population for Cost $C = 0.25$, $T = 5$, $R = 3$, $P = 1$, $S = 0$.

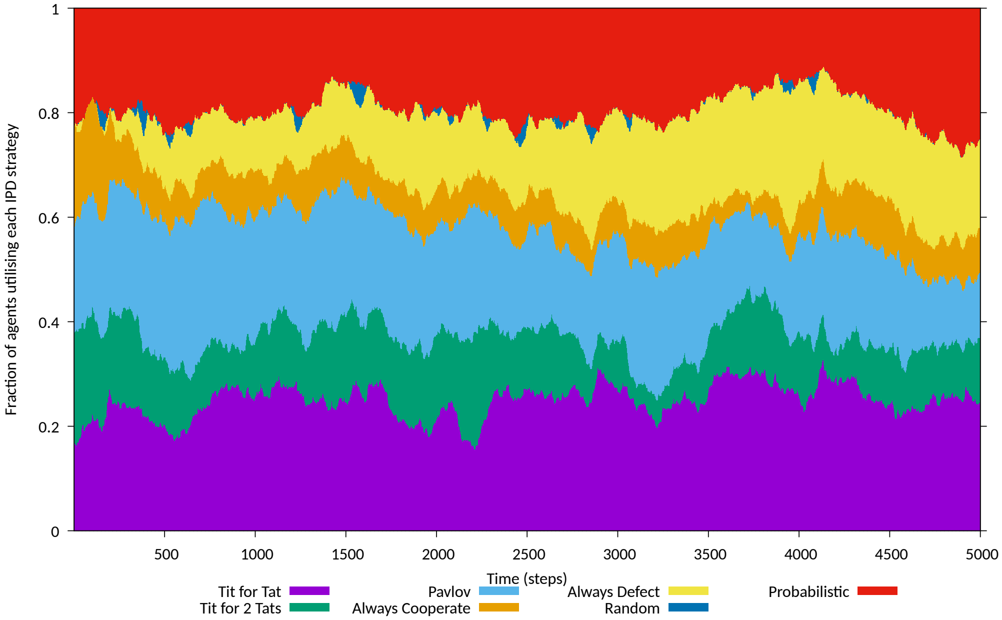

Fig. L. IPD Strategy Subpopulations for Cost $C = 0.25$, $T = 5$, $R = 3$, $P = 1$, $S = 0$.

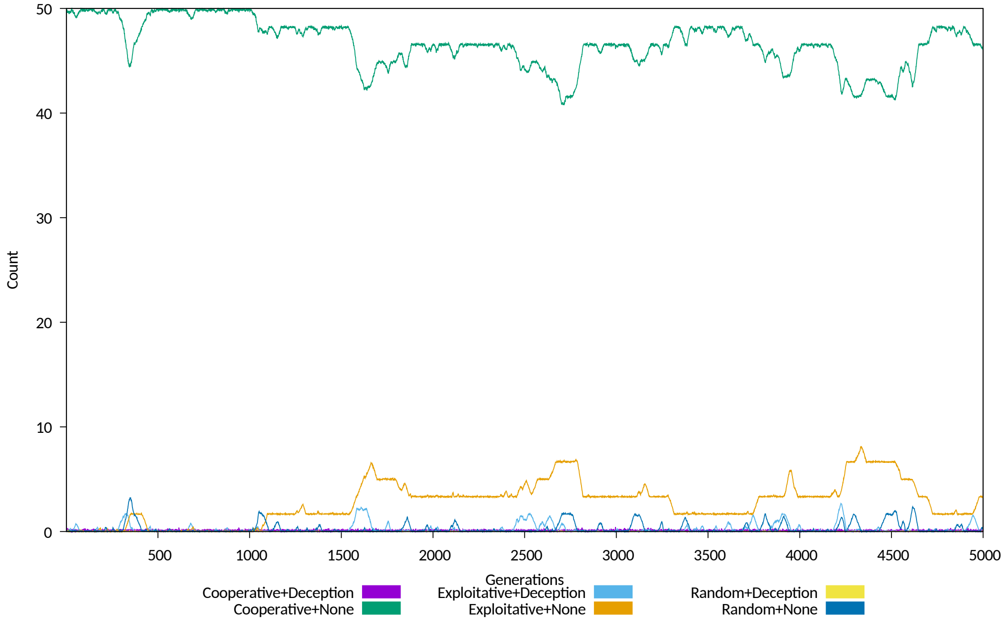

Fig. M. Degradation in Population for Cost $C = 0.3$, $T = 5$, $R = 3$, $P = 1$, $S = 0$.

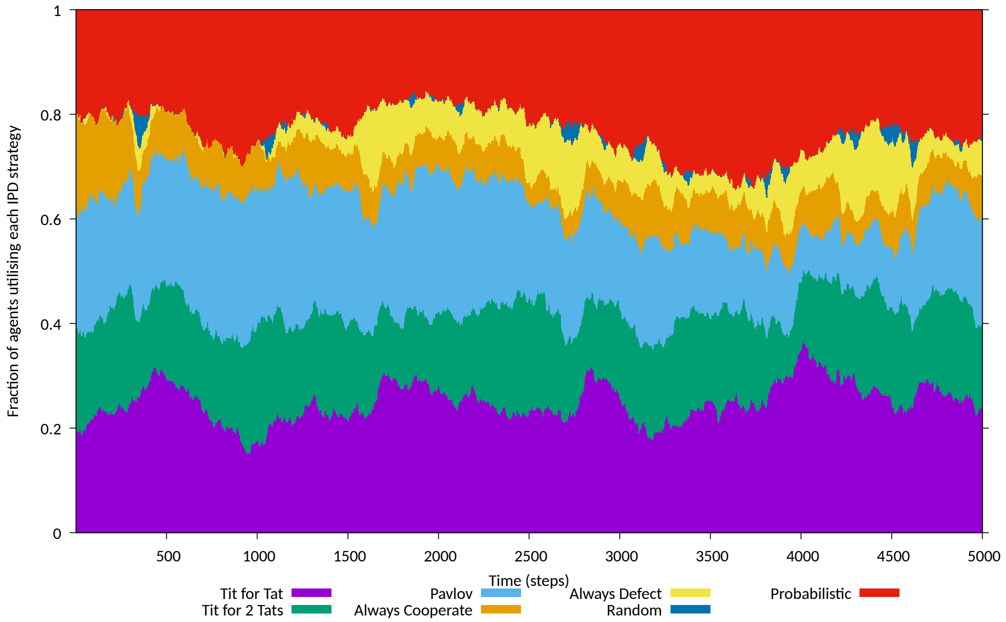

Fig. N. IPD Strategy Subpopulations for Cost $C = 0.3$, $T = 5$, $R = 3$, $P = 1$, $S = 0$.

Cost	Subpopulation	Mean	Sigma	Min	Max	Median
0.0	Cooperative Strategies + Deception	0.0012	0.0009	0.0000	0.0060	0.0013
0.0	Cooperative Strategies + No Deception	0.0086	0.0171	0.0000	0.0813	0.0007
0.0	Exploitative Strategies + Deception	0.7289	0.0332	0.6480	0.8040	0.7320
0.0	Exploitative Strategies + No Deception	0.2610	0.0268	0.1953	0.3420	0.2593
0.0	Random Strategy + Deception	0.0002	0.0004	0.0000	0.0027	0.0000
0.0	Random Strategy + No Deception	0.0001	0.0003	0.0000	0.0020	0.0000
0.05	Cooperative Strategies + Deception	0.0009	0.0012	0.0000	0.0093	0.0007
0.05	Cooperative Strategies + No Deception	0.1903	0.0476	0.0987	0.3007	0.1887
0.05	Exploitative Strategy + Deception	0.0150	0.0181	0.0000	0.0833	0.0067
0.05	Exploitative Strategy + No Deception	0.7900	0.0514	0.6773	0.8987	0.7967
0.05	Random Strategy + Deception	0.0001	0.0003	0.0000	0.0027	0.0000
0.05	Random Strategy + No Deception	0.0037	0.0078	0.0000	0.0373	0.0007
0.1	Cooperative Strategies + Deception	0.0007	0.0010	0.0000	0.0080	0.0007
0.1	Cooperative Strategies + No Deception	0.1703	0.0476	0.0660	0.2807	0.1693
0.1	Exploitative Strategy + Deception	0.0143	0.0193	0.0000	0.1207	0.0040
0.1	Exploitative Strategy + No Deception	0.8126	0.0526	0.6887	0.9327	0.8040
0.1	Random Strategy + Deception	0.0001	0.0002	0.0000	0.0027	0.0000
0.1	Random Strategy + No Deception	0.0019	0.0053	0.0000	0.0327	0.0000
0.15	Cooperative Strategies + Deception	0.0012	0.0011	0.0000	0.0080	0.0007
0.15	Cooperative Strategies + No Deception	0.4318	0.0719	0.2620	0.5627	0.4413
0.15	Exploitative Strategy + Deception	0.0157	0.0170	0.0000	0.0747	0.0087
0.15	Exploitative Strategy + No Deception	0.5488	0.0731	0.4127	0.7320	0.5327
0.15	Random Strategy + Deception	0.0001	0.0004	0.0000	0.0047	0.0000
0.15	Random Strategy + No Deception	0.0024	0.0061	0.0000	0.0340	0.0000
0.2	Cooperative Strategies + Deception	0.0013	0.0010	0.0000	0.0053	0.0013
0.2	Cooperative Strategies + No Deception	0.5059	0.0703	0.3953	0.6940	0.4980
0.2	Exploitative Strategy + Deception	0.0097	0.0139	0.0000	0.0813	0.0020
0.2	Exploitative Strategy + No Deception	0.4783	0.0647	0.3040	0.5693	0.4967
0.2	Random Strategy + Deception	0.0001	0.0003	0.0000	0.0033	0.0000
0.2	Random Strategy + No Deception	0.0047	0.0098	0.0000	0.0540	0.0007
0.25	Cooperative Strategies + Deception	0.0018	0.0012	0.0000	0.0093	0.0020
0.25	Cooperative Strategies + No Deception	0.8152	0.0327	0.7053	0.8947	0.8040
0.25	Exploitative Strategy + Deception	0.0061	0.0099	0.0000	0.0473	0.0007
0.25	Exploitative Strategy + No Deception	0.1736	0.0328	0.0987	0.2613	0.1693
0.25	Random Strategy + Deception	0.0000	0.0002	0.0000	0.0020	0.0000
0.25	Random Strategy + No Deception	0.0033	0.0067	0.0000	0.0327	0.0007
0.3	Cooperative Strategies + Deception	0.0019	0.0011	0.0000	0.0087	0.0020
0.3	Cooperative Strategies + No Deception	0.9142	0.0382	0.8153	0.9673	0.9287
0.3	Exploitative Strategy + Deception	0.0048	0.0089	0.0000	0.0533	0.0007
0.3	Exploitative Strategy + No Deception	0.0730	0.0318	0.0320	0.1627	0.0667
0.3	Random Strategy + Deception	0.0000	0.0002	0.0000	0.0013	0.0000
0.3	Random Strategy + No Deception	0.0061	0.0102	0.0000	0.0473	0.0007

Table S1. The *stable polymorphism* displays very close to equilibrium behaviour between the populations employing cooperative and exploitative strategies, but not employing the *Degradation* deception, between 1,500 to 2,000 generations, and 5,000 generations. The mean, standard deviation, minimum, maximum and median values are tabulated for the 1,999 to 4999 generations interval, for Cost values of 0.0, 0.05, 0.1, 0.15, 0.2, 0.25 and 0.3. Perturbations compared to a stable equilibrium arise as random mutations produce agents employing exploitative strategies, with or without deception, and these transiently invade the population. The special case of a Cost value of 0 produces a collapse to a *non-cooperative equilibrium* at ≈2,600 generations.
