# Supplementary material for: Information-theoretic models of deception: Modelling cooperation and diffusion in populations exposed to "fake news"
Source: PLoS One. 2018 Nov 28;13(11):e0207383. doi: 10.1371/journal.pone.0207383 (PMC6261583; doi:10.1371/journal.pone.0207383)
Supplement: S4 Appendix — These plots show the observed transient diffusion behaviour for exploitative strategies, comprising agents employing Always Defect, with or without the concurrent use of the Corruption deception, parametrised by cost. (PDF) [file pone.0207383.s004.pdf]

S4 Appendix

Cost Dependency of Differential Model Fit in Corruption Experiments

These plots show the observed transient diffusion behaviour for exploitative strategies, comprising agents employing *Always Defect*, with or without the concurrent use of the *Corruption* deception, parametrised by cost.

In a typical simulation run, multiple transient cycles occur, as agents in the populate mutate to employ the *Corruption* deception, gain a foothold in the population, increase in numbers, while agents employing *Always Defect* concurrently increase in numbers, eventually forcing agents that employ the *Corruption* deception out of the population, upon which agents employing cooperative strategies gain a fitness advantage and rapidly outcompete agents employing *Always Defect*.

The plots show a very close fit to the differential model, which is similar to the SIR epidemiological model widely employed in the modelling or analysis of diffusion in social media populations.

Differential Model Fitting to IPD Diffusion Behaviours

From the purely mathematical point of view, all the epidemiological compartment models considered are based on the physical model of a collection of agents who exclusively take on one of the possible traits. Using the principle of local conservation of agents, each agent has a rate of change of trait that depends on their trait and the population of all the other trait groups. For example, a cooperator might spontaneously convert to being a defector, or perhaps an defector might be convinced from meeting a cooperator to become a cooperator.

Each model, SIS, SEIZ, etc - is first order. The distribution of changes in trait populations is a function of the trait populations themselves. Let P be the tuple of population counts. $\Delta P = F(S)$. Injecting an explicit Δt, this is rephrased as $\Delta P = F(S)\Delta t$. Based on a Taylor approximation the following model is used:

$\Delta p_k = \lambda_k + \sum_j \beta_{jk} p_j + \sum_{ij} \mu_{ijk} p_i p_j$

Being the spontaneous generation (births, deaths, immigration, emigration) and the spontaneous conversion, and the induced conversions to trait i.

To pass to the continous, $s_k = p_k/N$ is used, which becomes a continous variable as $N \to \infty$. This has the advantage by design that the behaviour of the system is moderately immune to the actual population of the traits, depending only on the relative fractions. It is the physical assumption that as the population increases, the rate at which people meet people does not increase. The motivation is not to model an increase in population - but to model the existing population with a continous model in which the popualtion is infinite.

Assuming also that λ_k, β_{jk} and μ_{ijk} are rates of occurence of a point process, the model becomes:

$\dot{s}_k = \lambda_k + \sum_j \beta_{jk} p_j + \sum_{ij} \mu_{ijk} p_i p_j$

This covers all the models referenced, but also includes such things as an agnostic becoming an atheist after meeting with a theist. Unlike simple infection, a meeting can have twisted conscequences. Hence the mu is a rank-3 tensor and not rank-2 as is assumed in most of the models.

Given time series data $[s_k]_t$ where t is a discrete time of a discrete simulation. There is also $[\dot{s}_k]_t$ and $[s_i s_j]_t$ for each i, and j. The problem of model fitting is to determine $[\dot{s}_k]_t$ as

a linear combination of the $[s_k]_t$ and $[s_i s_j]_t$. If the number of traits is m, then there are m distinct s_k and $m(m+1)/2$ $s_i s_j$ including the square population vectors.

Placing these in a matrix F, and forming $U = F^T F$ and $V = F^T[\dot{s}]$, the optimal parameters are $A = U^{-1}V = (F^T F)^{-1} F^T [\dot{s}]$, assuming that F^T is of full rank, and the output of this model is $E = F^T A = F^T (F^T F)^{-1} F^T [\dot{s}]$, which if $F^T F$ is of full rank is readily seen to be $E = [\dot{s}]$, although this exact fit is not to be expected.

The result is presented below.

The fitted model, a differential model, produces behaviour that is qualitatively similar to the simulation - especially in locating the existence, position, and height of these spikes in derivative. Both the data and the fitted model conserve population to a high accuracy, about 5 decimal places, as testified by the last two images.

Results

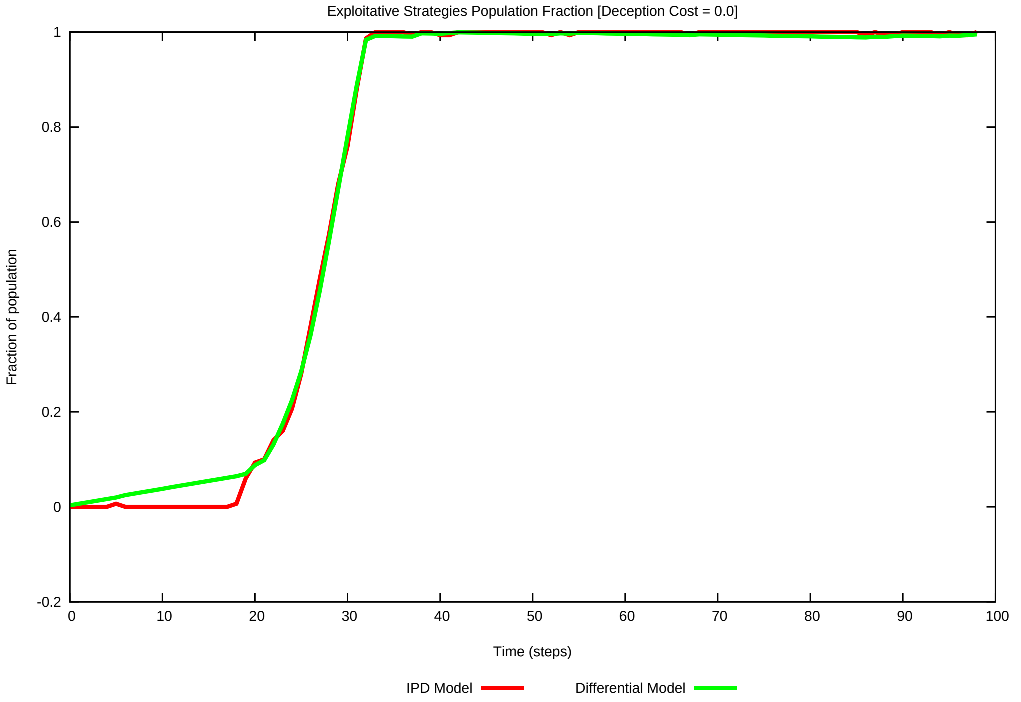

Fig. A. Differential model fit for $Cost = 0$, $T = 5$, $R = 3$, $P = 1$, $S = 0$.

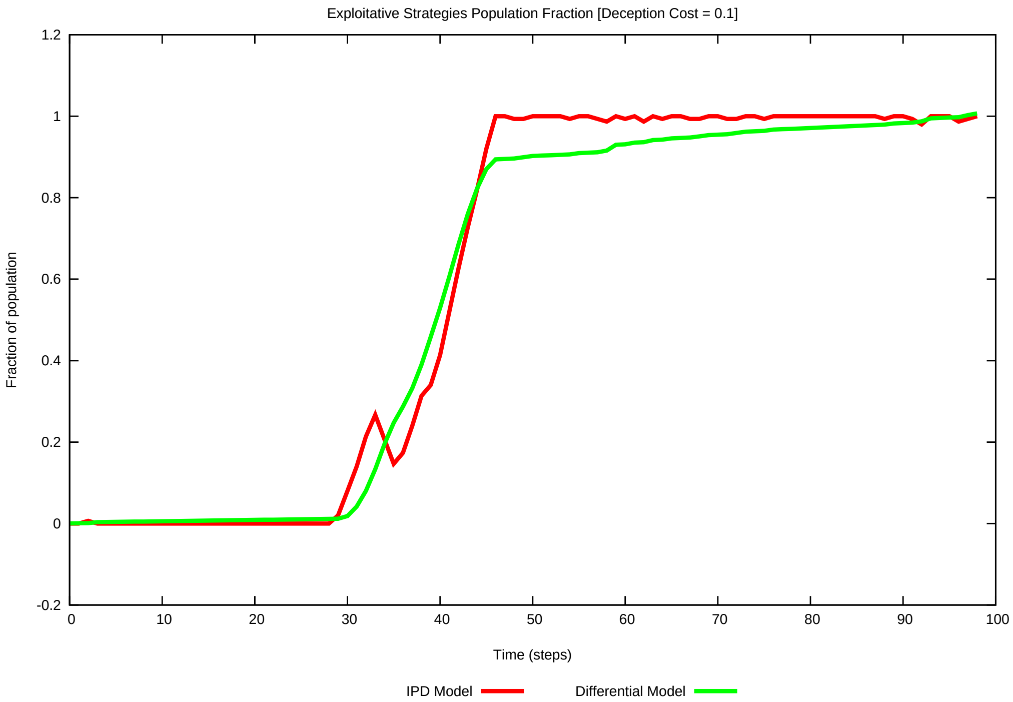

Fig. B. Differential model fit for $Cost = 0.1,\ T = 5,\ R = 3,\ P = 1,\ S = 0.$

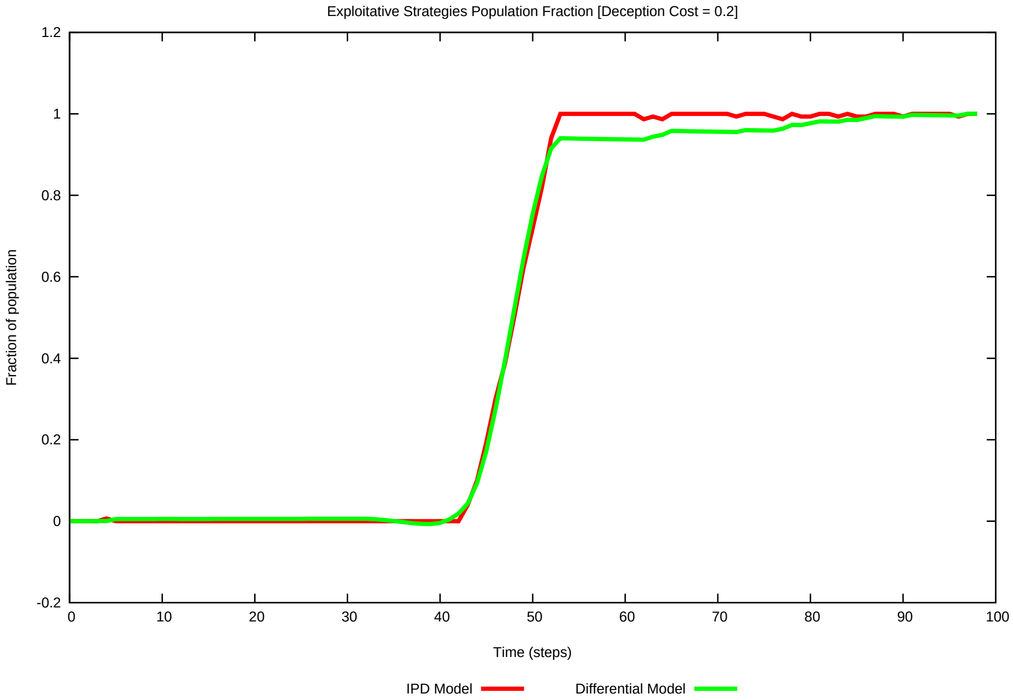

Fig. C. Differential model fit for $Cost = 0.2,\ T = 5,\ R = 3,\ P = 1,\ S = 0.$

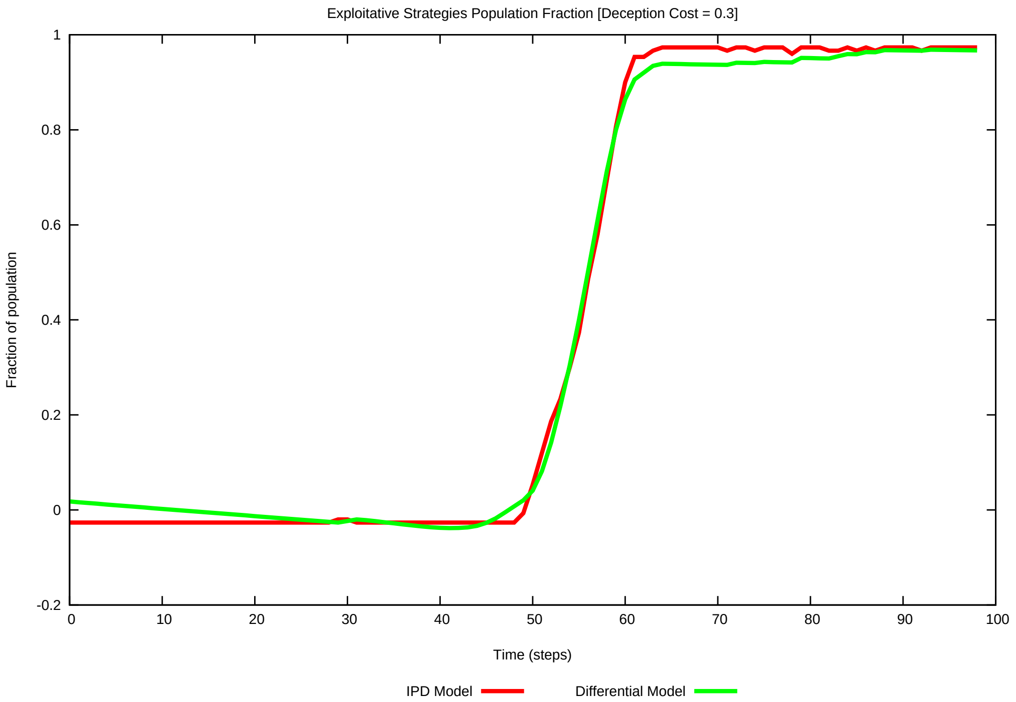

Fig. D. Differential model fit for $Cost = 0.3$, $T = 5$, $R = 3$, $P = 1$, $S = 0$.

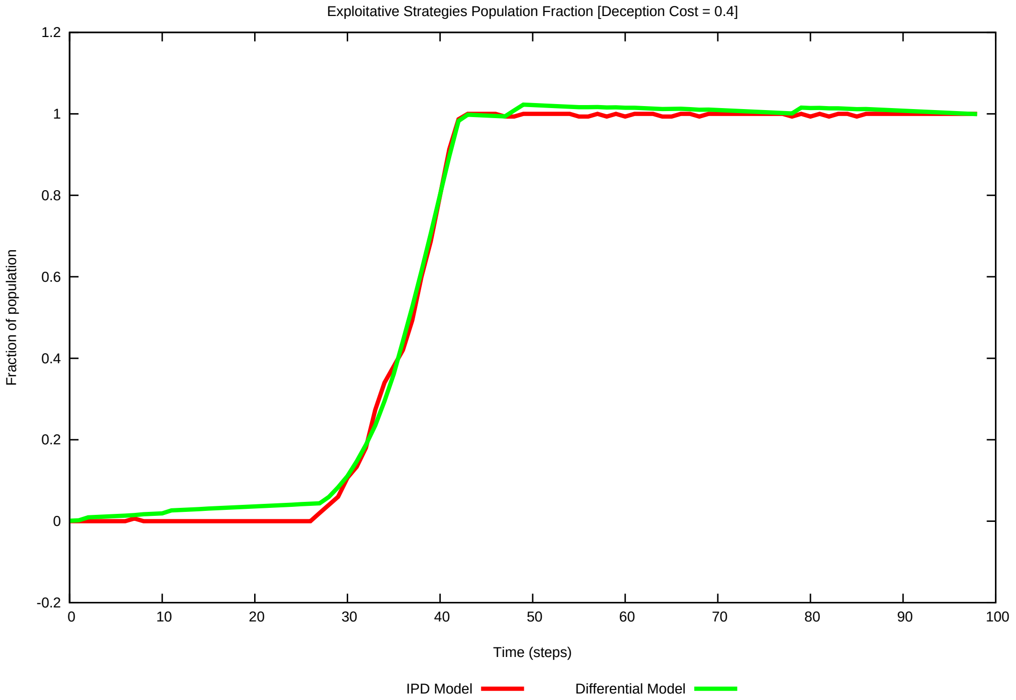

Fig. E. Differential model fit for $Cost = 0.4$, $T = 5$, $R = 3$, $P = 1$, $S = 0$.

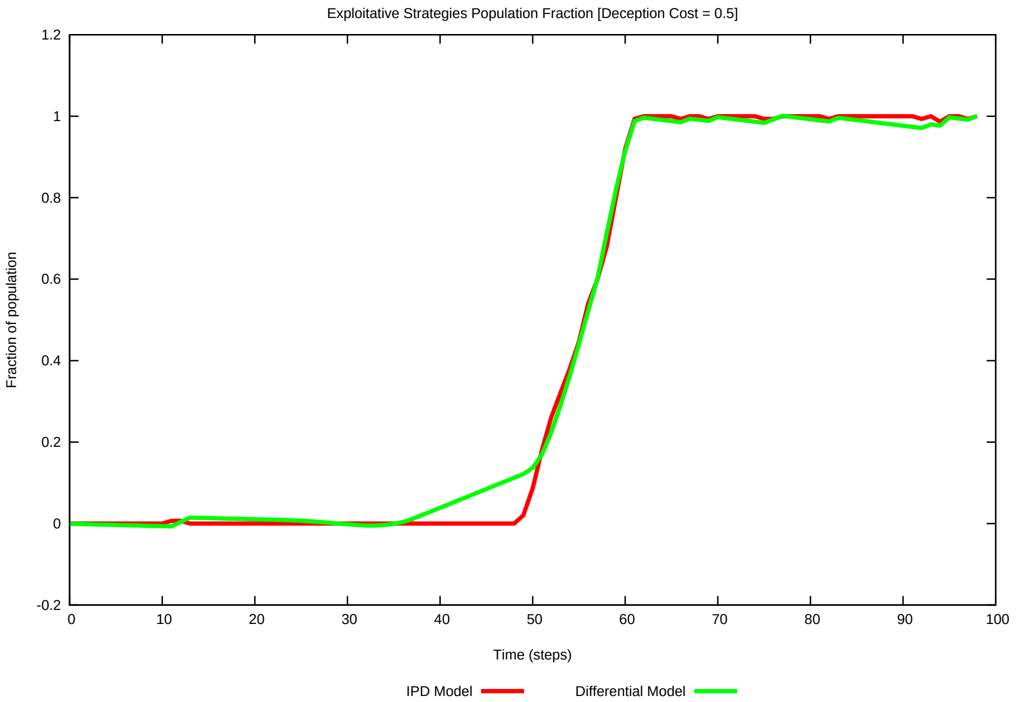

Fig. F. Differential model fit for $Cost = 0.5$, $T = 5$, $R = 3$, $P = 1$, $S = 0$.

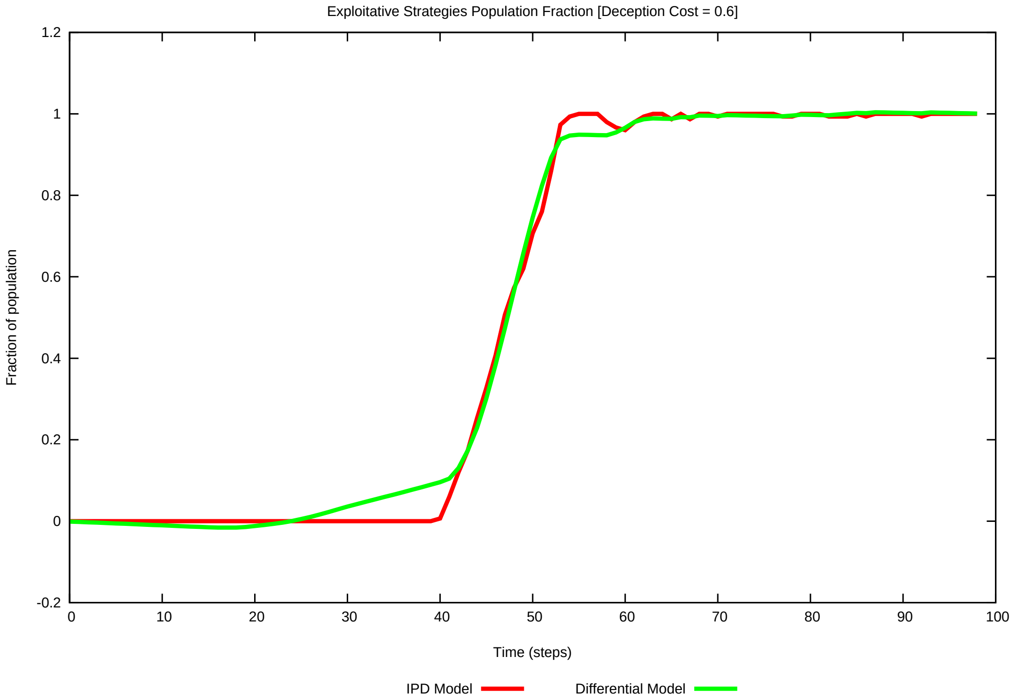

Fig. G. Differential model fit for $Cost = 0.6$, $T = 5$, $R = 3$, $P = 1$, $S = 0$.

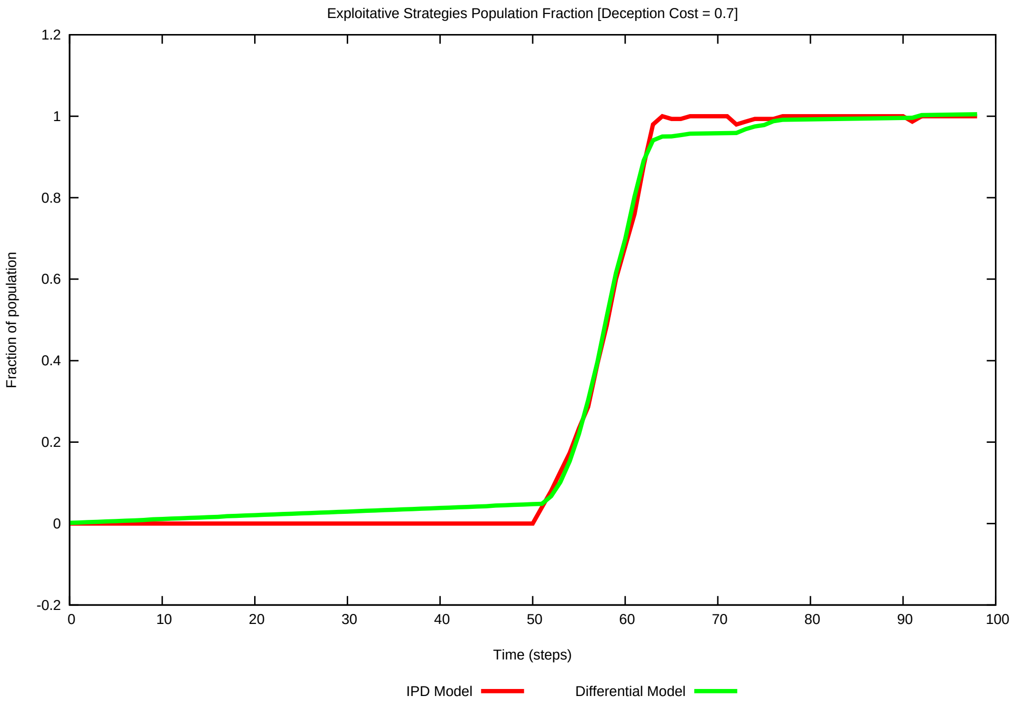

Fig. H. Differential model fit for $Cost = 0.7$, $T = 5$, $R = 3$, $P = 1$, $S = 0$.

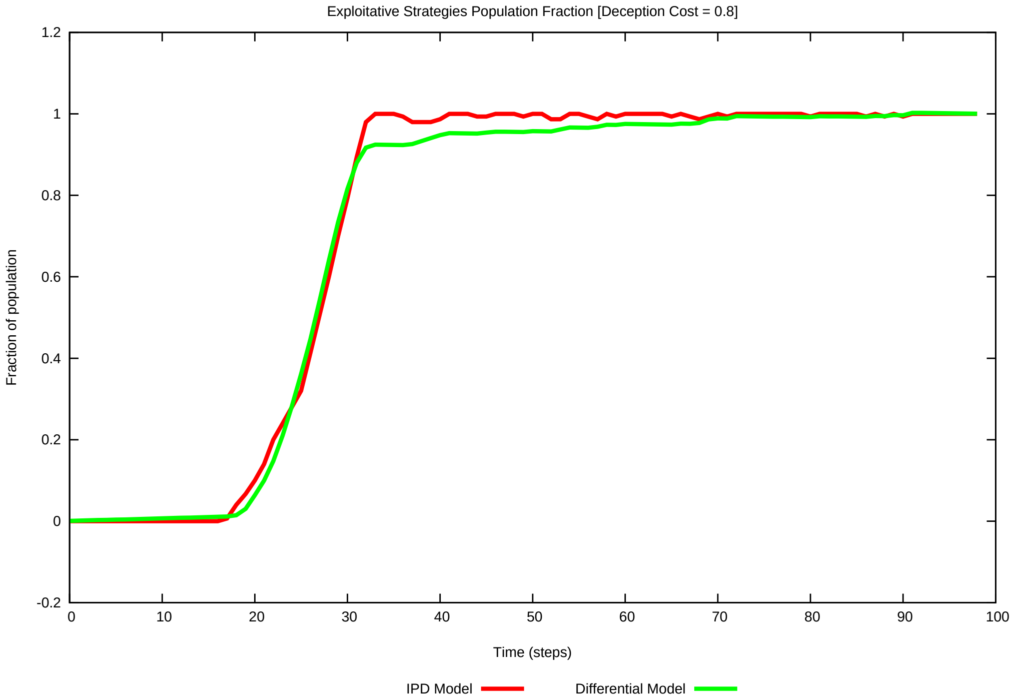

Fig. I. Differential model fit for $Cost = 0.8$, $T = 5$, $R = 3$, $P = 1$, $S = 0$.
